# Biomarkers of kidney involvement in paediatric IgA vasculitis: a systematic review and meta-analysis

**DOI:** 10.1007/s00467-026-07382-8

**Published:** 2026-06-05

**Authors:** Mădălina Andreea Beldie, Márk Viktor Hernádfői, Karen Krisztina Fazekas, Dóra Luca Bodócs, Péter Hegyi, Csaba Lódi, Iuliana Magdalena Stârcea

**Affiliations:** 1 Centre for Translational Medicine, Semmelweis University, Budapest, Hungary; 2 Grigore T. Popa, University of Medicine and Pharmacy, Iași, Romania; 3 Bethesda Children’s Hospital, Budapest, Hungary; 4 Pediatric Center, Semmelweis University, Budapest, Hungary; 5 Institute for Translational Medicine, Medical School, University of Pécs, Pécs, Hungary; 6 Institute of Pancreatic Diseases, Semmelweis University, Budapest, Hungary

**Keywords:** IgA vasculitis nephritis, Neutrophil-to-lymphocyte ratio (NLR), Platelet-to-lymphocyte ratio (PLR), Faecal occult blood test (FOBT), Biomarker, IgA vasculitis

## Abstract

**Background:**

IgA vasculitis nephritis (IgAVN) develops in 20–80% of patients with IgA vasculitis (IgAV), yet diagnosis is often delayed. Readily available biomarkers can help identify patients at risk earlier.

**Objective:**

To conduct a systematic review and meta-analysis of all available serum and urinary biomarkers to evaluate their prognostic potential in predicting IgAVN in paediatric patients.

**Data sources:**

A comprehensive search was performed in PubMed, EMBASE and Cochrane Library on November 9, 2024, covering publications from inception. The search strategy included three domains: paediatric population, IgAV diagnosis and studied biomarkers. No language or additional restrictions were applied.

**Study eligibility criteria and participants:**

We included original retrospective, prospective and cross-sectional studies that involved paediatric IgAV patients and reported biomarker levels in patients with and without nephritis. Two reviewers independently screened studies, and a third reviewer resolved conflicts.

**Study appraisal and synthesis methods:**

Data extraction was performed by two independent reviewers. A random-effects model was used for analysis. The review followed PRISMA guidelines, and study quality was assessed using the QUIPS tool. Outcomes were reported as mean or standardised mean differences with 95% confidence intervals (CI). Where possible, odds ratios (OR) and diagnostic performance measures were also evaluated.

**Results:**

Of 10,611 records screened, 129 studies including 23,726 patients were included. The neutrophil-to-lymphocyte ratio (NLR) was significantly higher in patients with nephritis (0.48; 95% CI 0.04–0.93), with notable effects in the Turkish population (0.86; 95% CI 0.19–1.52) but not in the Chinese population (− 0.02; 95% CI − 0.75–0.72). The platelet-to-lymphocyte ratio (PLR) showed a similar pattern, being higher in the Turkish subgroup (21.9; 95% CI 9.64–34.16) and lower in the Chinese subgroup (− 6.75; 95% CI − 11.27–2.22). A positive faecal occult blood test (FOBT) was associated with increased odds of kidney involvement (OR 2.04; 95% CI 1.05–3.96).

**Limitations:**

This study has limitations, including the predominance of retrospective data, limited reporting of diagnostic accuracy metrics (e.g. sensitivity, specificity), and substantial inter-study heterogeneity in nephritis definitions, measurement methods and study populations, which restrict firm conclusions about biomarker performance.

**Conclusions and implications of key findings:**

While statistically significant biomarker associations exist, clinically actionable predictors of IgAVN remain lacking. NLR, PLR and FOBT warrant prospective validation in multicentre cohorts with standardised protocols.

**Systematic review registration number:**

CRD42024593928.

**Graphical abstract:**

**Figure:**
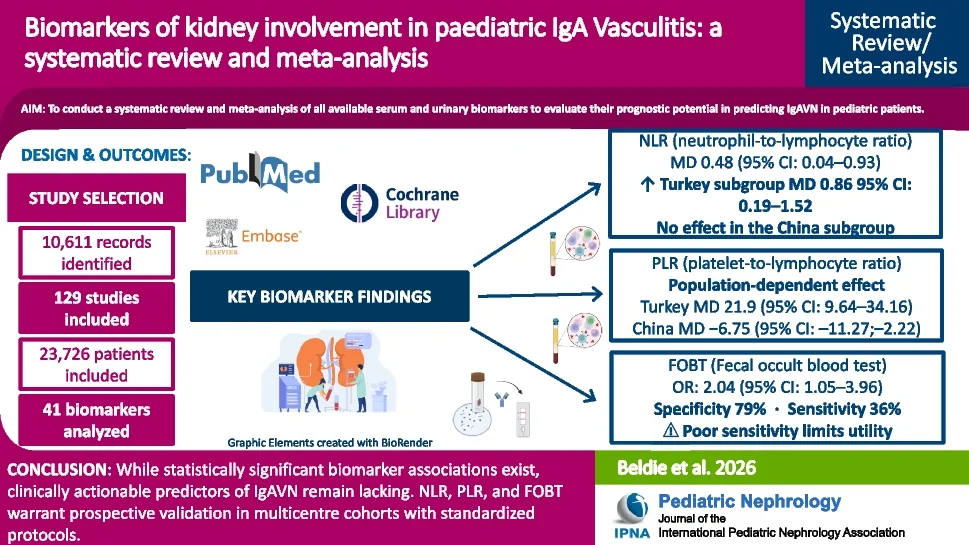
A higher resolution version of the Graphical abstract is available as Supplementary information

**Supplementary Information:**

The online version contains supplementary material available at https://doi.org/10.1007/s00467-026-07382-8.

## Introduction

IgA vasculitis (IgAV), formerly Henoch-Schönlein purpura, is the most common small-vessel vasculitis in children, with an incidence of 3–27 cases per 100,000, varying by geography [1]. The disease is driven by IgA-dominant immune complex deposition in small vessels, leading to systemic symptoms such as palpable purpura, gastrointestinal pain or bleeding, joint involvement and nephritis. Kidney involvement, known as IgA vasculitis nephritis (IgAVN), results from immune complex deposition in glomerular and interstitial vessels [2]. It plays a critical role in long-term prognosis, as persistent inflammation can lead to fibrosis and chronic kidney disease (CKD) [1, 3].

Nephritis occurs in 20–80% of IgAV cases and can present with a wide clinical spectrum, from isolated hematuria to nephrotic-range proteinuria or acute kidney injury, developing in the first 6 months after disease onset in 97% of cases. Although urinalysis remains the main tool for diagnosing nephritis in IgA vasculitis, biomarkers may help identify children at higher risk during the presymptomatic interval, rather than replacing routine urine monitoring [3].

Biomarkers have been explored as early indicators of kidney involvement. Previous systematic reviews have identified promising candidates such as kidney injury molecule-1 (KIM-1), platelet and leukocyte counts and complement factor 3 (C3). However, earlier analyses were limited by insufficient data, preventing strong conclusions or incorporation into clinical guidelines [4–6].

This study aimed to address those limitations by conducting a more comprehensive systematic review and meta-analysis of serum and urinary biomarkers associated with nephritis in paediatric IgAV. By evaluating a broader range of studies and populations, the goal was to strengthen the evidence for biomarker-based risk stratification and support the development of predictive tools for clinical practice.

## Methods

We conducted and reported our systematic review and meta-analysis following the current *Preferred Reporting Items for Systematic Reviews and Meta-Analyses* (PRISMA) guidelines [7] (see Supplementary Table S1) and the *Cochrane Handbook for Systematic Reviews* [8]. The study protocol was registered with the *International Prospective Register of Systematic Reviews* (PROSPERO; registration number CRD42024593928) [9] and the review was performed in accordance with the registered protocol. A detailed method description is presented in the Supplementary Methods section (Supplementary Page 1).

### Eligibility criteria

We included studies reporting on paediatric patients with IgAV aged 0–18 years and providing data on systemic involvement, particularly kidney involvement, and on blood or urinary biomarkers. Only original research articles, specifically, prospective and retrospective cohort studies that included patients with and without nephritis, were eligible. No restrictions were applied regarding follow-up duration or language of publication. We excluded non-original studies, such as reviews, case reports, case series and conference abstracts, as well as studies that exclusively involved adult patients, patients with kidney involvement only or studies that did not separately report data on patients with systemic involvement.

### Information sources

A comprehensive search was conducted on November 9, 2024, across three databases: MedLine (via PubMed), EMBASE and the Cochrane Central Register of Controlled Trials (CENTRAL). Studies were included from inception to the date of search. We further screened full-text articles through forward and backward citation chasing, using *Citation Chaser* [10] tool.

### Search strategy

In our search, we employed key terms related to the paediatric population, IgAV and biomarkers, grouping biomarkers into three domains: haematological, inflammatory and kidney injury-related markers. The detailed search key is provided in Supplementary Table S1.

### Selection process

Two independent reviewers (M.A.B. and D.L.B.) performed study selection after automatic and manual removal of duplicates using *EndNote* [11], first by title and abstract, then by full-text review, employing *Rayyan* [12]. Discrepancies were resolved through discussion with a third reviewer (M.H.). Cohen’s kappa coefficients were 0.95 for title-abstract screening and 0.96 for full-text selection, indicating almost perfect agreement.

### Data collection process

Two authors (M.A.B. and D.L.B.) independently conducted data extraction using an Excel spreadsheet (Office 365; Microsoft). When overlapping populations were identified across multiple studies, we included the study with the largest sample size. Articles published in languages other than English were translated using DeepL [13] or BeringAI [13] before data extraction. Data presented in the figures were extracted using the WebPlot Digitizer tool [14].

### Data items

Extracted data included first author, year of publication, study population, study period and design, country of study, follow-up duration, patient demographics, diagnostic definitions for IgAV and IgAV nephritis (IgAVN), and all reported biomarker data.

### Risk of bias assessment

Two authors (M.A.B. and D.L.B.) independently assessed the risk of bias using the *Quality in Prognostic Studies* (QUIPS) tool [15], with discrepancies resolved through discussion and consensus. The Cohen’s kappa for risk of bias assessment was 0.90. Visual summaries were generated using the Robvis tool [16].

### Synthesis methods

For dichotomous outcomes, the odds ratio (OR), and for continuous outcomes, the difference between the means (MD) or standardised mean difference (SMD) with a 95% confidence interval (CI) were used as effect size measures. To calculate the study odds ratios and the pooled odds ratio, we extracted the total number of patients and those with the event of interest in each group separately (referred to as “raw data”) or calculated them from the studies when available. We reported the results as the odds of an event of interest in the experimental group relative to the odds in the control group. In cases where only OR without “raw data” was given, we used the OR and its 95% confidence interval. For diagnostic outcomes, we extracted or calculated the counts of true positives, false positives, false negatives and true negatives.

To calculate the study (S)MDs and the pooled (S)MD, as well as the sample size, we extracted the mean and corresponding standard deviation (SD) from each study (in each group separately). We reported the results as experimental group minus control group values. To calculate the specificity and sensitivity, a joint analysis was performed.

Results were considered statistically significant if the pooled CI did not contain the null value.

Absolute between-study heterogeneity was expressed by tau, and relative between-study heterogeneity was described using Higgins and Thompson’s *I*^2^ statistics [17] for classical meta-analyses.

We reported the prediction intervals (i.e. the expected range of true effects in future studies) for the results if there were eight or more studies to be pooled.

Small study publication bias was assessed by visual inspection of Funnel plots and by calculating Harbord (modified Egger’s) test *p*-value [18, 19] for OR effect size, and classical Egger’s test *p*-value for MD and SMD effect sizes [20].

We planned to assume a possible small study bias if the *p*-value was less than 10% (although we kept in mind that the test had limited diagnostic assessment below ~ 10 studies).

Potential outlier publications were explored using different influence measures and plots following the recommendation of Harrer et al. [21].

All statistical analyses were made with R [22] using the meta [23] package for basic meta-analysis calculations and plots, and the *dmetar* [24] package for additional influential analysis calculations and plots, and *lme4* [25] package for diagnostic meta-analyses (for additional details on the synthesis method, see the Supplementary Material (Supplementary Page 1)).

## Results

### Search and selection

A total of 7512 records were identified through the systematic database search, and an additional 3099 through citation tracking. Of these, 78 studies [26–103] were included in the quantitative analysis, while all 129 were assessed in the systematic review. The detailed selection process is illustrated in the PRISMA flowchart (Supplementary Figure S1). For studies lacking sufficient data on patients with IgAVN, we contacted the corresponding authors to request individual patient data. After analysing their responses and the data provided in the articles, we included data from 7 additional papers [35, 38, 44, 50, 54, 71, 82] encompassing 636 patients.

### Basic characteristics of included studies

Our study, which analysed a combined total of 23,726 patients, provides a comprehensive overview of the current understanding of IgAVN. The majority of these patients were from Europe and the Western Pacific region. All studies included in our analysis were observational, of which 69 were prospective, 40 retrospective, 1 bidirectional and 6 cross-sectional studies. The baseline characteristics and the main findings of these studies are detailed in Supplementary Tables S2 and S3. In all cases, samples were collected at disease onset, prior to initiation of immunosuppressive treatment.

### Haematological and coagulation biomarkers

Haematological biomarkers are easily accessible and low-cost parameters that allow the identification of signs of systemic inflammation. The neutrophil-to-lymphocyte (NLR), platelet-to-lymphocyte (PLR) and mean platelet volume-to-platelet count (MPR) ratios have been increasingly used in cardiovascular diseases, neoplasia and autoimmune disorders [104–107].

Components of complete blood count (CBC) were evaluated both individually and as combined ratios. Data on coagulation biomarkers, specifically fibrinogen, D-dimers and activated partial thromboplastin time (APTT), were also assessed.

**Fig. 1:**
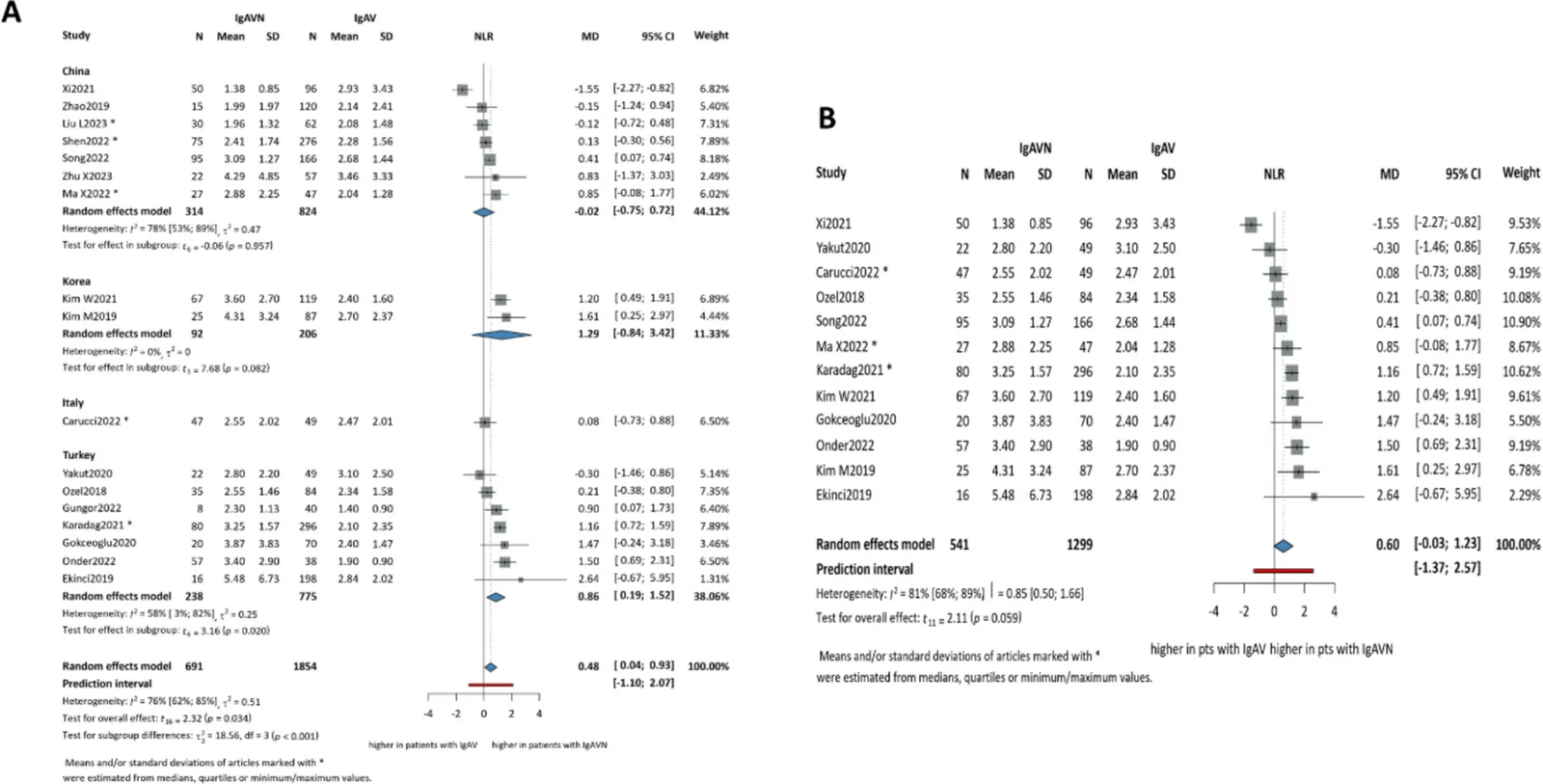
**A** MDs of NLR in patients with IgAV with and without renal involvement by country. **B** Sensitivity analysis including only the low risk of bias articles. Alt text: **A** Forest plot showing neutrophil-to-lymphocyte ratio differences between IgAVN and IgAV across countries, with subgroup and overall meta-analysis results. **B** Forest plot showing neutrophil-to-lymphocyte ratio differences between IgAVN and IgAV, including only the low risk of bias articles

**Fig. 2:**
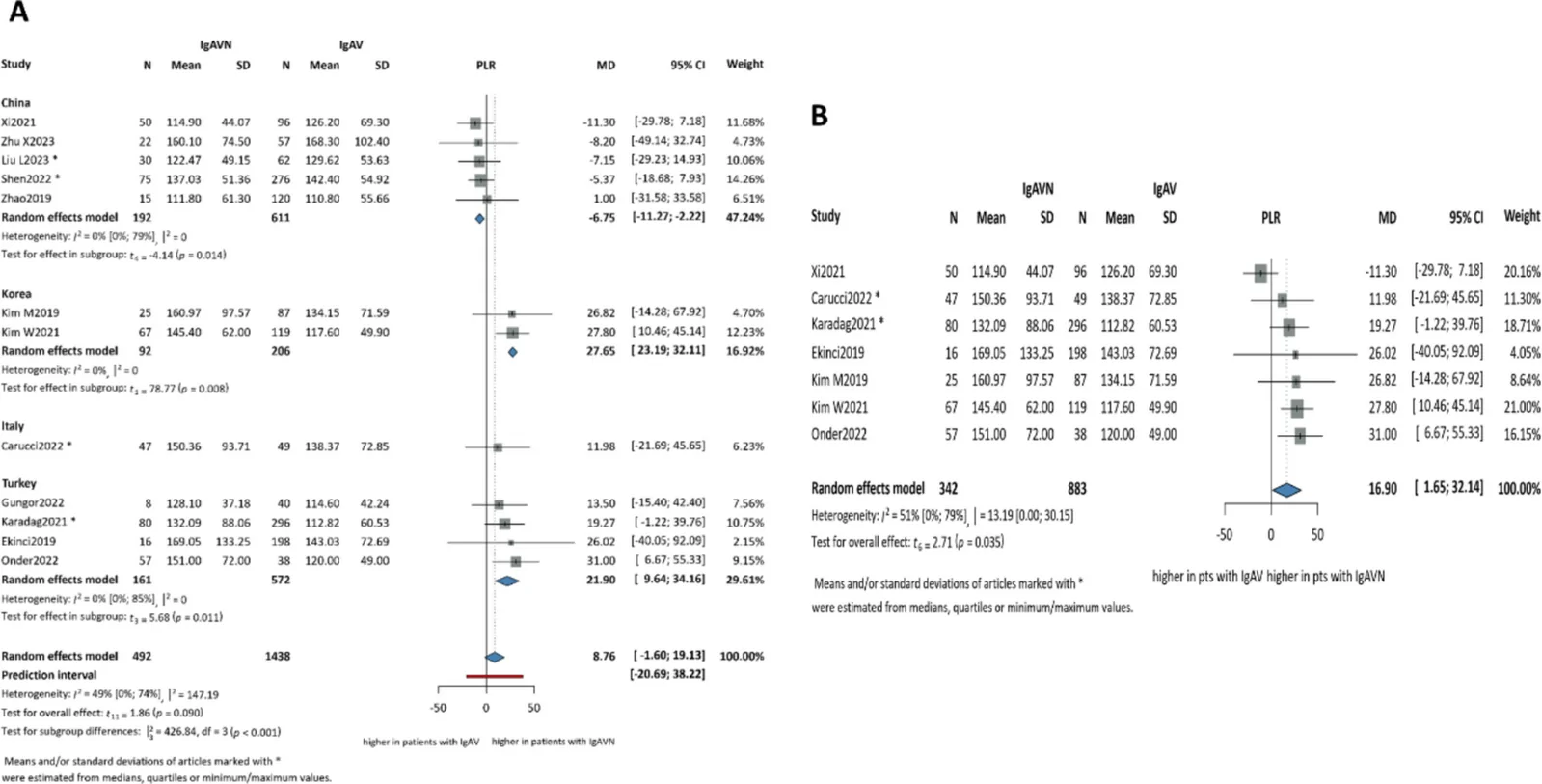
**A** MDs of PLR in patients with IgAV with and without renal involvement by country. **B** Sensitivity analysis including only the low risk of bias articles. Alt text: **A** Forest plot showing platelet-to-lymphocyte ratio differences between IgAVN and IgAV across countries, with subgroup and overall meta-analysis results. **B** Forest plot showing platelet-to-lymphocyte ratio differences between IgAVN and IgAV, including only the low risk of bias articles

Our analysis demonstrated that NLR (Fig. 1), as well as neutrophil and lymphocyte counts (Supplementary Figures S5 and S8), showed significant differences between patients with IgAV with and without nephritis, with statistically significant results in the group of studies conducted in Turkey (MD = 0.86, 95% CI [0.19; 1.52]). Furthermore, in the subgroup analysis by country of origin, PLR (Fig. 2) was significantly higher in Korea (MD = 27.65, 95% CI [23.10; 32.11]) and Turkey (MD = 21.9, 95% CI [9.64; 34.16]). In contrast, in the China subgroup, PLR values were higher among patients without kidney involvement (MD = − 6.75, 95% CI [− 11.27; − 2.22]). Other haematological and coagulation biomarkers did not show a clinically or statistically significant difference between patients with and without kidney involvement (Table 1).

**Table 1:** Summary of biomarkers included in the meta-analysis

Outcome	Unit	Studies (no.)	Patients (total no.; IgAVN; IgAV)			MD/SMD [95% CI]	OR [95% CI]	Heterogeneity [95% CI]	Clinical relevance	Statistical significance
Hematological biomarkers										
WBC (S. Figure 2)	× 10^9^/L	34	6560	2399	4161	0.45 [− 0.08; 0.98]		80% [72%; 85%]	No	No
WBC – elevated (S. Figure 3)		15	2291	787	1504		1.03 [0.8; 1.33]	71% [51%; 83%]	No	No
Ne count (S. Figure 5)	× 10^9^/L	15	2609	770	1839	0.92 [0.13; 1.70]		66% [41%; 80%]	Yes	Yes
Ne (S. Figure 6)	%	3	790	374	416	0.97 [− 9.64; 11.58]		71% [2%; 92%]	No	No
Ne count_elevated (S. Figure 7)		4	364	119	245		1.64 [0.38; 6.95]	61% [0%; 87%]	No	No
Ly (S. Figure 8)	× 10^9^/L	13	1892	506	1386	− 0.29 [− 0.52; − 0.06]		44% [0%; 71%]	Yes	Yes
Mo (S. Figure 9)	× 10^9^/L	5	642	214	428	0.00 [–0.14; 0.15]		54% [0%; 83%]	No	No
Eo (S. Figure 10)	× 10^9^/L	4	380	96	284	0.02 [− 0.04; 0.08]		5% [0%; 85%]	No	No
RBC (S. Figure 11)	× 10^6^/μL	6	957	260	697	0.02 [–0.07; 0.12]		10% [0%;77%]	No	No
Hb (S. Figure 12)	g/dl	21	4119	1541	2578	0.24 [0.09; 0.39]		54% [24%;72%]	No	Yes
Hb_decreased (S. Figure 13)		8	1858	671	1187		1.22 [0.89; 1.65]	41% [0%;74%]	No	No
MCV (S. Figure 15)	fl	7	1303	455	848	1.37 [− 1.09; 3.83]		86% [73%; 93%]	No	No
MCV_decreased (S. Figure 16)		4	891	313	578		1.47 [0.24; 8.95]	66% [0%; 88%]	No	No
RDW-CV (S. Figure 17)	%	9	2848	1139	1709	0.24 [− 0.09; 0.57]		73% [46%; 86%]	No	No
RDW-SD (S. Figure 18)	fl	4	524	167	357	1.33 [0.32; 2.34]		22% [0%; 88%]	No	Yes
PLT (S. Figure 19)	× 10^3^/μL	35	6537	2332	4205	− 2.68 [− 14.16; 8.80]		83% [77%; 87%]	No	No
PLT_elevated (S. Figure 20)		11	1661	618	1043		1.00 [0.82; 1.22]	70% [45%; 84%]	No	No
Plateletcrit (S. Figure 22)	%	3	415	152	263	0.00 [− 0.04; 0.05]		8% [0%; 90%	No	No
PDW (S. Figure 23)	fl	4	507	199	308	0.49 [− 0.37; 1.36]		68% [6%;89%]	No	No
MPV (S. Figure 24)	fl	20	3830	1203	2627	0.08 [− 0.03; 0.20]		35% [0%; 62%]	No	No
MPV_elevated (S. Figure 25)		5	1348	387	961	1.14 [0.89; 1.44]		57% [0%; 84]	No	No
MPR (S. Figure 26)		3	610	155	455	0.00 [− 0.01; 0.01]		74% [12%; 92%]	No	No
NLR_elevated (S. Figure 27)		6	1152	359	793		1.10 [0.73; 1.66]	79% [53%; 90%]	No	No
PLR_elevated (S. Figure 28)		5	824	236	588		1.19 [0.46; 3.09]	64% [0%;88%]	No	No
**Coagulation biomarkers**										
aPTT (S. Figure 29)	s	5	664	225	439	− 0.73 [− 2.46; 1.00]		54% [0%; 83%]	No	No
D-dimers (S. Figure 30)	mg/l	10	1628	631	997	− 0.25 [− 0.75; 0.26]		92% [87%; 95%]	No	No
D-dimers_elevated (S. Figure 31)		4	625	268	357		0.79 [0.02; 38.43]	95% [89%; 97%]	No	No
Fibrinogen (S. Figure 32)	g/l	8	1424	554	870	0.01 [− 0.21; 0.24]		57% [6%; 81%]	No	No
**Inflammatory biomarkers**										
CRP (S. Figure 33)	mg/l	28	6362	2306	4056	− 1.52 [− 5.79; 2.74]		83% [76%; 88%]	No	No
CRP_elevated (S. Figure 34)		20	4464	1460	3004		0.99 [0.85; 1.15]	68% [49%; 80%]	No	No
ESR (S. Figure 36)	mm/h	20	4900	1777	3123	0.1 [− 0.08; 0.28]		89% [84%; 92%]	No	No
ESR_elevated (S. Figure 37)		15	3609	1248	2288		1.14 [0.8; 1.63]	76% [60%; 85%]	No	No
IL-6 (S. Figure 39)	pg/ml	3	178	80	98	− 0.31 [− 4.84; 4.22]		96% [91%; 98%]	No	No
IL-8 (S. Figure 40)	pg/ml	3	202	105	97	− 0.71 [− 10.7; 9.28]		99% [97%; 99%]	No	No
IL-10 (S. Figure 41)	pg/ml	3	254	123	131	0.46 [− 1.52; 2.44]		91% [78%; 97%]	No	No
TNFα (S. Figure 42)	pg/ml	3	202	105	97	3.44 [− 11.46; 18.33]		99% [98%; 99%]	No	No
**Immunological biomarkers**										
ASOT (S. Figure 43)	IU/ml	5	712	148	564	* Median difference 20.32 [− 7.26; 47.9]		0% [0%; 79%]	No	No
Positive ASOT (S. Figure 44)		8	1100	316	784		1.34 [0.78; 2.30]	28% [0%; 68%]	No	No
C3 (S. Figure 46)	g/l	22	3984	1478	2506	− 0.21 [− 0.40; − 0.01]		76% [65%; 84%]	No	Yes
C3_decreased (S. Figure 47)		9	2515	983	1532		0.84 [0.33; 2.18]	67% [32%; 83%]	No	No
C3_elevated (S. Figure 48)		4	450	148	302	1.18 [0.44; 3.18]		38% [0%; 79%]	No	No
C4 (S. Figure 50)	g/l	18	3426	1247	2179	− 0.26 [− 0.58; 0.06]		87% [81%; 91%]	No	No
C4_decreased (S. Figure 51)		3	282	110	172		5.55 [1.26; 24.58]	0% [0%; 90%]	No	Yes
C4_elevated (S. Figure 52)		4	772	231	541		0.96 [0.21; 4.36]	66% [1%; 88%]	No	No
IgA (S. Figure 54)	g/l	28	4335	1738	2597	0.27 [− 0.11; 0.65]		89% [85%; 92%]	No	No
IgA_elevated (S. Figure 55)		12	2086	737	1349		1.10 [0.86; 1.41]	33% [0%; 66%]	No	No
IgE (S. Figure 57)	IU/ml	6	1489	690	799	− 0.07 [− 0.26; 0.13]		35% [0%; 74%]	No	No
IgG (S. Figure 58)	g/l	22	3923	1654	2269	− 0.37 [− 0.75; 0.01]		93% [90%; 95%]	Yes	No
IgG_elevated (S. Figure 59)		7	1965	787	1178		0.85 [0.76; 0.96]	58% [2%; 82%]	Yes	Yes
IgM (S. Figure 61)	g/l	21	3801	1604	2197	0.11 [− 0.07; 0.29]		71% [55%; 81%]	No	No
IgM_elevated (S. Figure 62)		4	700	240	460		0.76 [0.17; 3.33]	58% [0%; 86%]	No	No
**Kidney injury-related biomarkers**										
Albumin (S. Figure 63)	g/l	12	2940	1168	1772	− 2.13 [− 4.18; − 0.08]		97% [96%; 98%]	No	Yes
Albumin_decreased (S. Figure 64)		6	675	216	458		0.92 [0.83; 1.03]	19% [0%; 63%]	No	No
Cholesterol (S. Figure 66)	mmol/L	4	2035	874	1161	0.46 [− 0.26; 1.18]		91% [79%; 96%]	Yes	No
CysC (S. Figure 67)	mg/L	5	1316	692	624	0.03 [− 0.42; 0.47]		85% [68%; 93%]	No	No
uβ2MG (S. Figure 68)	mg/L	4	460	193	267	1.15 [− 1.70; 3.99]		98% [96%; 99%]	No	No
**Miscellaneous Biomarkers**										
ET-1 (S. Figure 69)	ng/l	3	108	52	56	0.45 [− 0.84; 1.75]		57% [0%; 88%]	No	No
Vitamin D (S. Figure 70)	ng/ml	3	957	168	789	− 0.66 [− 1.63; 0.31]		73% [10%; 92%]	No	No

A sensitivity analysis restricted to low-risk-of-bias studies was performed for NLR and PLR. The low-risk subgroup analyses showed directionally consistent findings with the main analyses, although the NLR effect was attenuated and heterogeneity remained substantial, while PLR remained positively associated with nephritis (Figs. 1 and 2).

### Immunological and inflammatory biomarkers

The pathophysiology of the disease has a strongly immune-related component, as aberrant IgA production is a promoter of the disease. Moreover, the immune complex deposition can contribute to cytokine production and systemic inflammation [2]. Immunological biomarkers analysed included immunoglobulins (IgA, IgG, IgM, IgE), interleukins (IL-6, IL-8, IL-10), complement proteins (C3, C4) and tumour necrosis factor α (TNFα), along with traditional inflammatory markers, such as C-reactive protein (CRP) and erythrocyte sedimentation rate (ESR) (Table 1).

Our findings show that in the Chinese subgroup, IgG values (Supplementary Figure S58) were lower in patients with IgAVN, compared to those without kidney involvement (SMD − 0.43, 95% CI [− 0.66; − 0.20]), whereas in the studies from the European region, no clinically or statistically significant difference could be found. Other immunological biomarkers did not prove differences between patients with and without IgAVN.

Neither CRP (Supplementary Figure S33) nor ESR (Supplementary Figure S36) showed statistically or clinically relevant differences (MD − 1.52, 95% CI [− 5.79; 2.74] and SMD 0.10, 95% CI [− 0.08; 0.28], respectively).

### Kidney injury-related biomarkers

Evidence on kidney injury biomarkers in IgAVN remains limited. We included studies reporting serum cystatin C, urinary β2-microglobulin (uβ2MG) and indirect markers such as serum albumin and total cholesterol (Table 1).

Among these, serum albumin (Supplementary Figure S63) showed a modest but statistically significant decrease in IgAVN patients (MD − 2.13, 95% CI [− 4.18; − 0.08]), with no clinical relevance.

### Additional biomarkers

Biomarkers not categorised above included faecal occult blood test (FOBT), vitamin D and endothelin-1 (Table 1).

**Fig. 3:**
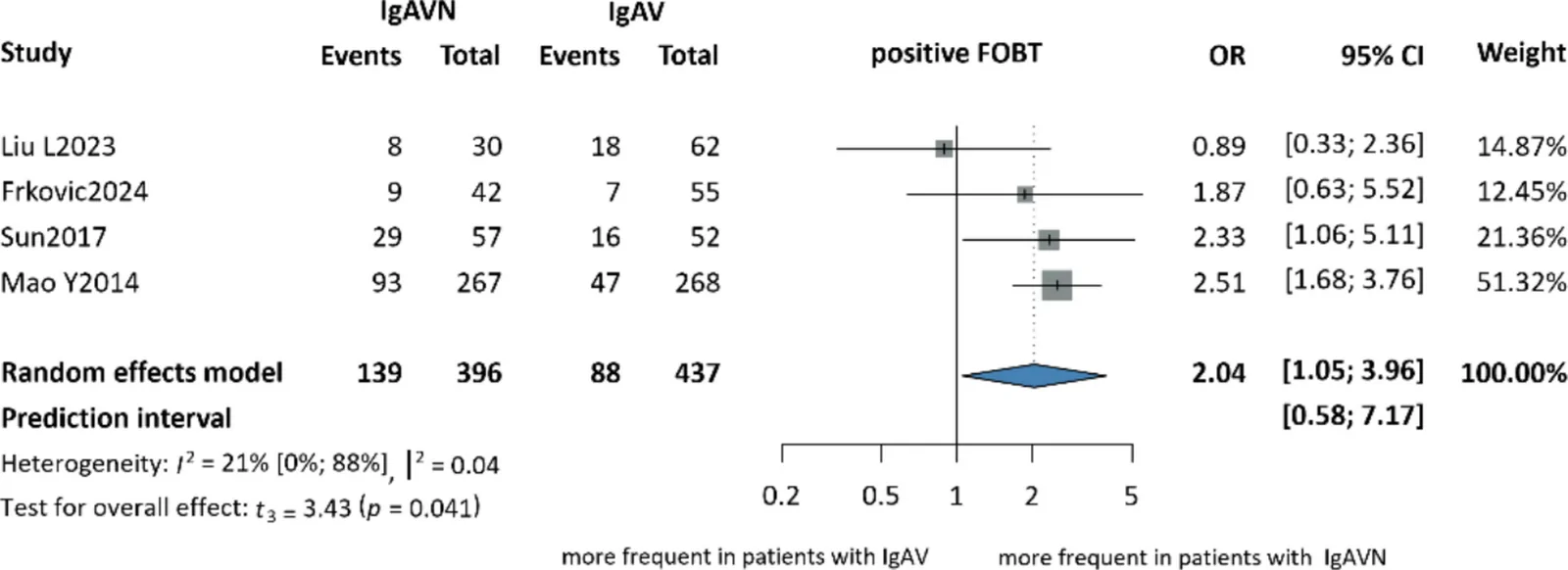
ORs of having a positive FOBT in patients with IgAV with and without renal involvement. Alt text: Forest plot displaying odds ratios for positive faecal occult blood test in IgAVN versus IgAV patients with pooled meta-analysis result

FOBT (Fig. 3) was analysed in several studies, comprising 396 IgAVN and 437 IgAV patients. A pooled OR of 2.04, 95% CI [1.05; 3.96] indicated increased odds of a positive test in patients with kidney involvement. However, diagnostic meta-analysis (Supplementary Figure S71) showed moderate specificity (79%) but low sensitivity (36%), suggesting that although FOBT may help exclude kidney involvement, its poor sensitivity limits its utility as a standalone screening tool. In this case, the number of low-risk studies was insufficient to support a robust separate pooled analysis.

The analysis of vitamin D and endothelin-1 (Supplementary Figures S69 and S70) failed to detect any significant differences between patients with and without kidney involvement.

### Diagnostic test meta-analysis

Using diagnostic performance parameters extracted from the included articles, we conducted a diagnostic test meta-analysis for 12 biomarkers (haemoglobin, WBC, PLT, antistreptolysin O titre, C3, C4, CRP, ESR, IgA, IgG, albumin and FOBT), none of which demonstrated reliable performance as predictive standalone biomarkers (Supplementary Figures S4, S14, S21, S35, S38, S45, S49, S53, S56, S60, S65, S71).

### Qualitative analysis

Our systematic review included 129 studies reporting on 121 clinical and pre-clinical biomarkers, both urine- and serum-derived. Markers such as matrix metalloproteinase-9 [108] (MMP-9), monocyte chemoattractant protein-1 [109] (MCP-1) and faecal calprotectin [56] showed promising results in identifying patients at risk for kidney involvement (Supplementary Table S2).

### Risk of bias assessment

Of the included studies, 58 (44.96%) were rated as low risk of bias, 66 (51.16%) as moderate risk and 5 (3.87%) as high risk. The most common source of bias was related to outcome measurement, specifically the lack of a transparent or standardised definition of kidney involvement. Additional issues included unclear inclusion criteria, absence of follow-up information, and incomplete reporting of statistical methods. Assessments are detailed in Supplementary Figure S74 and Tables S4–7.

### Publication bias and heterogeneity

Regional differences (as reflected in subgroup analyses) and possibly ethnicity contributed substantially to heterogeneity, particularly in haematological biomarkers. Despite these adjustments, a substantial portion of heterogeneity remained unexplained.

Potential publication bias was observed for WBC and PLT, although Egger’s test results may be due to high heterogeneity across large studies (Supplementary Figures S72, S73). No publication bias was identified for other outcomes.

## Discussion

This study aimed to identify and analyse serum and urinary biomarkers with a predictive role for kidney involvement in paediatric patients with IgAV. Our synthesis encompasses over 23,000 patients, from 129 studies and provides the most comprehensive and methodologically rigorous evaluation to date of predictive biomarkers for IgAVN. While some haematological and clinical biomarkers demonstrated statistically significant associations with kidney involvement, their clinical utility remains limited due to limited data on diagnostic performance and considerable inter-study heterogeneity.

### Haematological and coagulation biomarkers

Among haematological markers, NLR and PLR emerged as some of the most frequently evaluated and potentially useful indices. These ratios, which reflect shifts in leukocyte populations during inflammatory responses, are considered to provide a more robust reflection of systemic inflammation than individual white blood cell (WBC) counts alone [110]. In subgroup analyses, both NLR and PLR were significantly elevated in patients with kidney involvement in studies conducted in Turkey and Korea. However, this association was not replicated in the Chinese subgroup, where PLR values were higher in patients without kidney involvement. These discrepancies raise critical questions about the consistency and generalizability of haematological biomarkers across populations and the role of genetic background in the pathogenesis of the disease [111].

Several studies have attempted to validate NLR and PLR as systemic inflammatory markers in IgAV. Although Jaszczura et al. showed that NLR is a particularly appropriate biomarker for triage of patients at risk of systemic involvement during the course of IgAV, Ekinci et al., Kim et al. and Onder et al. concluded that higher NLR levels are associated with kidney and severe gastrointestinal involvement [41, 59, 72, 110]. Additionally, Onder et al. also acknowledged the role of PLR and platelet-related indices in identifying patients at risk for kidney involvement [72].

Nevertheless, although these associations are statistically significant, they lack standardised thresholds and are characterised by wide inter-study variability. Our meta-analysis supports the interpretation that, although NLR and PLR may offer clinical insights into systemic inflammation, their standalone application as predictive tools for IgAVN is currently not supported. Similarly, data from a cohort of IgA Nephropathy (IgAN) suggest that these markers perform well at distinguishing between patients with IgAN and healthy controls, but they are not able to predict patient outcomes [112].

Moreover, lower levels of MPV have been highlighted as a marker of poor prognosis in IgAN [112], possibly due to the association of MPV with chronic inflammation. However, our meta-analysis did not replicate this result in IgAVN, especially because samples were collected at disease onset and chronic inflammation had not yet been established.

### Gastrointestinal involvement and the use of FOBT

Gastrointestinal (GI) involvement in IgAV, including abdominal pain, haematochezia and positive FOBT, has previously been associated with an increased risk of kidney involvement. In our review, FOBT was associated with a pooled odds ratio (OR) of 2.04 [1.05;3.96] for the presence of nephritis. However, diagnostic performance parameters revealed only moderate specificity (79%) and poor sensitivity (36%), limiting its potential as a reliable screening tool.

Sestan et al. found in a Croatian multicenter cohort that IgAVN may be predicted by older age, the presence of GI symptoms at disease onset and the severity of GI involvement [113]. Additionally, the meta-analysis of Chan et al. [4] found no difference in the severity of GI involvement when examining the association with kidney involvement. Clinically, this suggests that paediatric patients with early GI signs, particularly those with a positive FOBT, may require more aggressive nephrologic surveillance, even in the absence of established nephritis. Multiple studies also identified elevated NLR and PLR values as common predictors of both kidney and GI involvement, reinforcing the conclusion that these manifestations may arise from similar inflammatory pathways [41, 57, 72, 82, 92].

### Immunological and inflammatory biomarkers

In our meta-analysis, we also examined a range of immunological and inflammatory biomarkers, including immunoglobulin subclasses (IgA, IgG, IgM), interleukins (IL-6, IL-8, IL-10), complement proteins (C3, C4), TNF-α and traditional markers such as CRP and ESR. Overall, none of these markers showed consistent, clinically significant differences between IgAV patients with and without nephritis. A notable exception was IgG, which appeared to be lower in nephritis patients in the Chinese cohort, a finding not replicated in European or other Western Pacific populations.

The pathophysiological mechanisms underlying reduced IgG in nephritis are not fully understood but may involve several mechanisms. Suzuki et al. suggest that in patients with IgAV without kidney involvement, immune complexes contain IgA but no IgG, whereas in patients with IgAVN and IgAN, immune complexes contain IgA and IgG [114]. This supports the hypothesis that IgG may be consumed in immune complex formation and deposited in kidney tissue, resulting in lower circulating levels [111]. However, this mechanism remains speculative in the context of IgAV and requires further investigation, particularly across ethnically and geographically diverse cohorts.

### Kidney injury-related biomarkers

Among kidney injury–related markers, we identified only a limited number of studies evaluating serum cystatin C [47, 63, 64, 75] and uβ2MG [47, 63, 64, 75]. It is worth noting that many of the most promising biomarker candidates were identified in small, single-centre studies or lacked standardised measurement and analysis protocols. Although early findings suggested potential utility for detecting subclinical kidney injury, data are currently too sparse to allow definitive conclusions. Future studies should employ rigorous methodological designs and prioritise reproducibility across centres and populations.

### Strengths and limitations

This review has several important strengths. It is the most comprehensive analysis of prognostic biomarkers for IgAVN to date, encompassing over 23,000 paediatric patients and 121 biomarkers. Our inclusion of studies from four continents enhances the generalizability of our findings and allows us to examine regional variability, a key factor given the known geographic differences in IgAV incidence and outcomes.

Nonetheless, this study is not without limitations. A significant proportion of the included studies were retrospective, increasing the risk of selection and information bias. Moreover, few studies reported diagnostic accuracy metrics such as sensitivity, specificity or area under the receiver operating characteristic curve (AUC), thereby limiting our ability to draw firm conclusions about the predictive power of individual biomarkers. The high degree of inter-study heterogeneity, particularly in definitions of nephritis (Supplementary Table S3), measurement methods, study populations and data reporting, also complicates interpretation.

Although biomarker samples were generally collected at disease onset and before treatment initiation, timing was not fully uniform across all studies. Differences in the interval from symptom onset, variation in gastrointestinal involvement and broader systemic inflammatory burden may have influenced biomarker levels and contributed to heterogeneity across analyses.

In addition, the rigorous methodology we employed is another strength of our study, as it enhances the robustness of our results.

### Implications for practice and research

The findings of this review provide a valuable foundation for future research. Biomarkers such as NLR, PLR and FOBT are inexpensive, non-invasive and widely available, and our results suggest they warrant further investigation as components of multivariate risk models. Future studies should focus on validating these markers in prospective, multicentre cohorts with standardised definitions and protocols.

Given the regional and possibly ethnic variability observed in biomarker performance, future research should also aim to account for population-specific genetic and environmental factors. Personalised risk prediction tools incorporating clinical variables and biomarker panels may offer a more feasible path forward than reliance on any single biomarker.

In addition, one of the most critical sources of heterogeneity in this review was the inconsistent definition of kidney involvement across studies. Establishing consensus-based diagnostic criteria and outcome measures for IgAVN should be a top priority for the research community. Such standardisation will be essential to enable meta-analyses, cross-study comparisons and the development of clinical decision support tools.

Furthermore, the identification of markers associated with kidney inflammation also supports the development of novel therapies or the repurposing of existing drugs. A better understanding of the disease's pathophysiology represents a step forward in the discovery of treatments to prevent IgAVN. In this manner, the goal of reducing the burden of CKD in IgAVN patients becomes attainable.

## Conclusion

Elevated NLR, PLR and positive FOBT were associated with kidney involvement in IgA vasculitis in selected populations, suggesting their potential but still limited utility. These findings support future prospective studies and the development of predictive models for diverse clinical settings and paediatric populations.

## Supplementary Information

Below is the link to the electronic supplementary material.

Graphical abstract (PPTX 292 KB)

Supplementary file (DOCX 7.63 MB)

## Data Availability

The datasets analysed during the current study are not publicly available but are available from the corresponding author on reasonable request.
