# Supplementary material for: Biomarkers of kidney involvement in paediatric IgA vasculitis: a systematic review and meta-analysis
Source: Pediatr Nephrol. 2026 Jun 5;41(12):4379–92. doi: 10.1007/s00467-026-07382-8 (PMC13672260; doi:10.1007/s00467-026-07382-8)
Supplement: Supplementary file 1 [file 467_2026_7382_MOESM2_ESM.docx]

| PRISMA Checklist | 2-4 |
| --- | --- |
| Data Synthesis | 5 |
| Detailed Search Strategy | 6-10 |
| Supplementary Tables and Figures | 11-64 |
| Risk of bias assessment | 65-69 |
| References | 70 |

**Table of contents**

| **Section and Topic** | **Item #** | **Checklist item** | **Location where item is reported** |
| --- | --- | --- | --- |
| **TITLE** | | |  |
| Title | 1 | Identify the report as a systematic review. | 1 |
| **ABSTRACT** | | |  |
| Abstract | 2 | See the PRISMA 2020 for Abstracts checklist. | 3 |
| **INTRODUCTION** | | |  |
| Rationale | 3 | Describe the rationale for the review in the context of existing knowledge. | 5 |
| Objectives | 4 | Provide an explicit statement of the objective(s) or question(s) the review addresses. | 5 |
| **METHODS** | | |  |
| Eligibility criteria | 5 | Specify the inclusion and exclusion criteria for the review and how studies were grouped for the syntheses. | 6 |
| Information sources | 6 | Specify all databases, registers, websites, organisations, reference lists and other sources searched or consulted to identify studies. Specify the date when each source was last searched or consulted. | 6 |
| Search strategy | 7 | Present the full search strategies for all databases, registers and websites, including any filters and limits used. | 6 |
| Selection process | 8 | Specify the methods used to decide whether a study met the inclusion criteria of the review, including how many reviewers screened each record and each report retrieved, whether they worked independently, and if applicable, details of automation tools used in the process. | 6 |
| Data collection process | 9 | Specify the methods used to collect data from reports, including how many reviewers collected data from each report, whether they worked independently, any processes for obtaining or confirming data from study investigators, and if applicable, details of automation tools used in the process. | 6 |
| Data items | 10a | List and define all outcomes for which data were sought. Specify whether all results that were compatible with each outcome domain in each study were sought (e.g. for all measures, time points, analyses), and if not, the methods used to decide which results to collect. | 6 |
|  | 10b | List and define all other variables for which data were sought (e.g. participant and intervention characteristics, funding sources). Describe any assumptions made about any missing or unclear information. | 6 |
| Study risk of bias assessment | 11 | Specify the methods used to assess risk of bias in the included studies, including details of the tool(s) used, how many reviewers assessed each study and whether they worked independently, and if applicable, details of automation tools used in the process. | 6 |
| Effect measures | 12 | Specify for each outcome the effect measure(s) (e.g. risk ratio, mean difference) used in the synthesis or presentation of results. | 7, Supplementary Page 1 |
| Synthesis methods | 13a | Describe the processes used to decide which studies were eligible for each synthesis (e.g. tabulating the study intervention characteristics and comparing against the planned groups for each synthesis (item #5)). | 7 |
|  | 13b | Describe any methods required to prepare the data for presentation or synthesis, such as handling of missing summary statistics, or data conversions. | 7 |
|  | 13c | Describe any methods used to tabulate or visually display results of individual studies and syntheses. | 7 |
|  | 13d | Describe any methods used to synthesize results and provide a rationale for the choice(s). If meta-analysis was performed, describe the model(s), method(s) to identify the presence and extent of statistical heterogeneity, and software package(s) used. | 7, Supplementary Page 1 |
|  | 13e | Describe any methods used to explore possible causes of heterogeneity among study results (e.g. subgroup analysis, meta-regression). | 7, Supplementary Page 1 |
|  | 13f | Describe any sensitivity analyses conducted to assess robustness of the synthesized results. | 7, Supplementary Page 1 |
| Reporting bias assessment | 14 | Describe any methods used to assess risk of bias due to missing results in a synthesis (arising from reporting biases). | 6 |
| Certainty assessment | 15 | Describe any methods used to assess certainty (or confidence) in the body of evidence for an outcome. | - |
| **RESULTS** | | |  |
| Study selection | 16a | Describe the results of the search and selection process, from the number of records identified in the search to the number of studies included in the review, ideally using a flow diagram. | 7, Supplementary Figure 1 |
|  | 16b | Cite studies that might appear to meet the inclusion criteria, but which were excluded, and explain why they were excluded. |  |
| Study characteristics | 17 | Cite each included study and present its characteristics. | 7, Supplementary Table 2 |
| Risk of bias in studies | 18 | Present assessments of risk of bias for each included study. | 9, Supplementary Figure 74, Supplementary Table 4-7 |
| Results of individual studies | 19 | For all outcomes, present, for each study: (a) summary statistics for each group (where appropriate) and (b) an effect estimate and its precision (e.g. confidence/credible interval), ideally using structured tables or plots. | 8-9, Supplementary Figures 2-71 |
| Results of syntheses | 20a | For each synthesis, briefly summarise the characteristics and risk of bias among contributing studies. | 8-9, Supplementary Figures 2-71 |
|  | 20b | Present results of all statistical syntheses conducted. If meta-analysis was done, present for each the summary estimate and its precision (e.g. confidence/credible interval) and measures of statistical heterogeneity. If comparing groups, describe the direction of the effect. | 8-9, Supplementary Figures 2-71 |
|  | 20c | Present results of all investigations of possible causes of heterogeneity among study results. | 8-9, Supplementary Figures 2-71 |
|  | 20d | Present results of all sensitivity analyses conducted to assess the robustness of the synthesized results. | 8-9, Supplementary Figures 2-71 |
| Reporting biases | 21 | Present assessments of risk of bias due to missing results (arising from reporting biases) for each synthesis assessed. | 9, Supplementary Figure 74, Supplementary Table 4-7 |
| Certainty of evidence | 22 | Present assessments of certainty (or confidence) in the body of evidence for each outcome assessed. | - |
| **DISCUSSION** | | |  |
| Discussion | 23a | Provide a general interpretation of the results in the context of other evidence. | 10-11 |
|  | 23b | Discuss any limitations of the evidence included in the review. | 11 |
|  | 23c | Discuss any limitations of the review processes used. | 11 |
|  | 23d | Discuss implications of the results for practice, policy, and future research. | 11 |
| **OTHER INFORMATION** | | |  |
| Registration and protocol | 24a | Provide registration information for the review, including register name and registration number, or state that the review was not registered. | 6 |
|  | 24b | Indicate where the review protocol can be accessed, or state that a protocol was not prepared. | 6 |
|  | 24c | Describe and explain any amendments to information provided at registration or in the protocol. | 6 |
| Support | 25 | Describe sources of financial or non-financial support for the review, and the role of the funders or sponsors in the review. | 2 |
| Competing interests | 26 | Declare any competing interests of review authors. | 2 |
| Availability of data, code and other materials | 27 | Report which of the following are publicly available and where they can be found: template data collection forms; data extracted from included studies; data used for all analyses; analytic code; any other materials used in the review. |  |

*From:* Page MJ, McKenzie JE, Bossuyt PM, Boutron I, Hoffmann TC, Mulrow CD, et al. The PRISMA 2020 statement: an updated guideline for reporting systematic reviews. BMJ 2021;372:n71. doi: 10.1136/bmj.n71. This work is licensed under CC BY 4.0. To view a copy of this license, visit <https://creativecommons.org/licenses/by/4.0/>

**Methods – Data Synthesis**

In case the SD was not given, but instead the SEM was available, the SD was calculated as SEM multiplied by the square root of the sample size. If instead of the mean, SD or SEM the quartiles were given, for estimating the mean and standard deviation from the quartiles Lou^1^ and Shi^2^ methods were used (as implemented in the used _meta_ R package). As a limitation, we should highlight that this is an estimation.

Although based on the other publications in the literature about these outcomes and the data in the used publication, we could assume that the distribution of this variable does not relevantly differ from a normal or log-normal distribution, therefore this estimation might give only a small bias. For the outcomes that we had IPD, we also looked at the data to assess their distribution.

The inverse variance weighting method was used to calculate the pooled OR (based on directly given OR without "raw data"), MD and SMD. We used a Hartung-Knapp adjustment ^3,4^ for CIs.

Hedges' g (bias corrected standardized mean difference; using exact formulae): Hedges^5^ calculated the exact bias in Cohen's d, which is a ratio of gamma distributions with the degrees of freedom, i.e. total sample size minus two, as argument. The exact formulae are used to calculate the standardized mean difference as well as the standard error.

To estimate the heterogeneity variance measure τ^2^ for all of the outcomes, the restricted maximum-likelihood estimator was used with the Q profile method for confidence interval^6,7^.

On the forest plots, the t-distribution-based method was used for the CI of MD calculation of individual studies. The exact method used for CI of SMD calculation of individual studies. In case of 0 cell counts, individual study OR with type 95% CI was calculated by adding 0.5 as continuity correction (it was used only for visualization on the forest plot). We assumed different τ^2^ values in the subgroups.

To assess the difference between the subgroups, a “Cochrane Q” test (an omnibus test) was used between subgroups^6^. The null hypothesis was rejected on a 5% significance level.

In case of 24 variables, the subgroup analysis was decided after data extraction as the data implied this structure.

For diagnostic outcomes, at first, we extracted or calculated the numerical data of true positive, false positive, false negative and true negative counts (referred as “raw counts”), if it was available. The calculations of counts required no assumptions; these calculations were based on the sample size, other diagnostic test parameters, and additional available data in the articles.

A joint analysis of specificity and sensitivity was carried out using a bivariate binomial Generalized Linear Mixed Model based on the article Chu and Cole^8^. This analysis takes into account the dependency between specificity and sensitivity. The calculations were based on the code of the online tool described in the article Freeman et al.^9^. It assumes "each study varies but comes from a common underlying distribution with an unstructured between-study covariance matrix" and it uses "the delta method to calculate the standard errors of the likelihood ratios and diagnostic odds ratio to allow calculation of the confidence intervals" ^9^.

We presented the results on ROC plots, which show the specificity and sensitivity values with the corresponding 95% prediction regions at the study level, as well as the summary estimate of specificity and sensitivity and the corresponding 95% confidence region yielded by the fitted model (not reported). We also created forest plots, where we presented the extracted or calculated raw counts, the calculated sensitivity and specificity values with their exact binomial (95%) confidence intervals for each study. In this plot, the estimate of pooled sensitivity and specificity values based on the bivariate model was also visualized. Heterogeneity of between-study effects was assessed visually based on the aforementioned prediction regions and the forest plots as recommended by Lee et al.^10^

**Detailed search strategy**

**Pubmed**

(neonat* OR pediatric* OR paediatric* OR "adolescent" OR "adolescence" OR "adolescency" OR child* OR "young" OR "young adult" OR "young adults" OR "kids" OR "youth" OR "juvenile" OR "juvenility" OR infant* OR "infancy" OR preschooler* OR "teen" OR "teens" OR teenager*)

AND

((("Henoch" AND ("Schonlein" OR "Schönlein")) OR "Henoch-Schonlein" OR "Henoch-Schönlein" OR "HSP" OR "HSPN" OR ("IgA" AND "vasculitis") OR "IgAV" OR "IgAVN" OR ("anaphylactoid" OR "allergic") AND "purpura"))

AND
("WBC" OR "leukocyte count" OR "NLR" OR "neutrophil-to-lymphocyte ratio" OR "neutrophil to lymphocyte ratio" OR "PLT" OR ”MPV” OR ”mean platelet volume” OR ”RDW” OR ”red cell distribution width” OR ”ESR” OR ”erythrocyte sedimentation rate” OR "C-reactive protein" OR "CRP" OR "FIBRINOGEN" OR "Fib" OR ”DD” OR ”D-dimers” OR ”serum sulfatide” OR ”erythrocyte glutathione S-transferase” OR e-GST OR "IgA" OR "immunoglobulin A" OR "IgG" OR "Immunoglobulin G" OR ”C3” OR ”C4” OR "Antiphospholipid antibodies" OR "APLA" OR "APLAs" OR "Anticardiolipin antibodies" OR "Anti-beta-2-glycoprotein-I antibod*" OR "anti-β2GPI antibod*" OR "Lupus anticoagulant" OR "lupus coagulation inhibitor" OR "Apolipoprotein M" OR "apoM" OR ”Pentraxin 3” OR ”PTX 3” OR ”MMP-9” OR ”MMP 9” OR ”matrix metalloproteinase-9” OR ”TIMP-1” OR ”TIMP 1” OR ” tissue inhibitor matrix metalloproteinase-1” OR ”MMP-9/TIMP-1” OR ” MMP-9/TIMP-1 ratio” OR "ACRU" OR "albumin creatinine ratio" OR "UACR" OR ”KIM-1” OR ”KIM 1” OR ”kidney injury molecule – 1” OR ”kidney injury molecule 1” OR ”NAG” OR ”N-acetyl-β-D-glucosamidase” OR ”NGAL” OR ”Neutrophil gelatinase-associated lipocalin” OR ”MCP-1” OR ”MCP 1” OR ”monocyte chemotactic protein-1” OR ”monocyte chemotactic protein 1” OR ”CysC” OR ”cystatin C” OR CD4 OR biomarker* OR (("biological" OR "molecular") AND "marker") OR "prognostic" OR "prognosis" OR "predictive")

Domain 1 refers to: pediatric population

Domain 2 refers to: IgA vasculitis, with synonyms – Henoch Schonlein purpura, anaphylactoid purpura, allergic purpura

Domain 3 refers to: biomarkers that can predict the onset of renal involvement

Number of results: 2952

Notes: I used the truncations for words describing pediatric population to find all varieties of the words – neonate, neonates, neonatal, or pediatric, pediatrics and so on. I used the quotation mark for the biomarkers so I can find those exact biomarkers I am searching for.

Domain 3 subgroups:

**Hematological and inflammatory markers:** "WBC" OR "leukocyte count" OR "NLR" OR "neutrophil-to-lymphocyte ratio" OR "neutrophil to lymphocyte ratio" OR "PLT" OR ”MPV” OR ”mean platelet volume” OR ”RDW” OR ”red cell distribution width” OR ”ESR” OR ”erythrocyte sedimentation rate” OR "C-reactive protein" OR "CRP" OR "FIBRINOGEN" OR "Fib" OR ”DD” OR ”D-dimers” OR ”serum sulfatide” OR ”erythrocyte glutathione S-transferase” OR e-GST

**Immunological tests:** "IgA" OR "immunoglobulin A" OR "IgG" OR "Immunoglobulin G" OR ”C3” OR ”C4” OR "Antiphospholipid antibodies" OR "APLA" OR "APLAs" OR "Anticardiolipin antibodies" OR "Anti-beta-2-glycoprotein-I antibod*" OR "anti-β2GPI antibod*" OR "Lupus anticoagulant" OR "lupus coagulation inhibitor"

**Renal damage specific markers:** "Apolipoprotein M" OR "apoM" OR ”Pentraxin 3” OR ”PTX 3” OR ”MMP-9” OR ”MMP 9” OR ”matrix metalloproteinase-9” OR ”TIMP-1” OR ”TIMP 1” OR ” tissue inhibitor matrix metalloproteinase-1” OR ”MMP-9/TIMP-1” OR ” MMP-9/TIMP-1 ratio” OR "ACRU" OR "albumin creatinine ratio" OR "UACR" OR ”KIM-1” OR ”KIM 1” OR ”kidney injury molecule – 1” OR ”kidney injury molecule 1” OR ”NAG” OR ”N-acetyl-β-D-glucosamidase” OR ”NGAL” OR ”Neutrophil gelatinase-associated lipocalin” OR ”MCP-1” OR ”MCP 1” OR ”monocyte chemotactic protein-1” OR ”monocyte chemotactic protein 1” OR ”CysC” OR ”cystatin C” OR CD4

Database settings: I searched for All fields, with no other special settings.

**Embase**

(neonat* OR pediatric* OR paediatric* OR adolescent OR adolescence OR adolescency OR child* OR young OR "young adult" OR "young adults" OR kids OR youth OR juvenile OR juvenility OR infant* OR infancy OR preschooler* OR teen OR teens OR teenager*)

AND

((Henoch AND (Schonlein OR Schönlein)) OR Henoch-Schonlein OR Henoch-Schönlein

OR HSP OR HSPN OR (IgA vasculitis) OR (IgA vasculitis nephritis) OR IgAV OR IgAVN OR (anaphylactoid AND purpura) OR (allergic AND purpura))

AND
(WBC OR "leukocyte count" OR NLR OR "neutrophil-to-lymphocyte ratio" OR "neutrophil to lymphocyte ratio" OR PLT OR MPV OR ”mean platelet volume” OR RDW OR ”red cell distribution width” OR ESR OR ”erythrocyte sedimentation rate” OR "C-reactive protein" OR CRP OR FIBRINOGEN OR Fib OR DD OR D-dimers OR ”serum sulfatide” OR ”erythrocyte glutathione S-transferase” OR e-GST OR IgA OR "immunoglobulin A" OR IgG OR "Immunoglobulin G" OR C3 OR C4 OR "Antiphospholipid antibodies" OR APLA OR APLAs OR "Anticardiolipin antibodies" OR "Anti-beta-2-glycoprotein-I antibod*" OR "anti-β2GPI antibod*" OR "Lupus anticoagulant" OR "lupus coagulation inhibitor" OR "Apolipoprotein M" OR apoM OR ”Pentraxin 3” OR ”PTX 3” OR ”MMP-9” OR ”MMP 9” OR ”matrix metalloproteinase-9” OR ”TIMP-1” OR ”TIMP 1” OR ” tissue inhibitor matrix metalloproteinase-1” OR ”MMP-9/TIMP-1” OR ” MMP-9/TIMP-1 ratio” OR ACRU OR "albumin creatinine ratio" OR UACR OR KIM-1 OR ”KIM 1” OR ”kidney injury molecule – 1” OR ”kidney injury molecule 1” OR NAG OR ”N-acetyl-β-D-glucosamidase” OR NGAL OR ”Neutrophil gelatinase-associated lipocalin” OR MCP-1 OR ”MCP 1” OR ”monocyte chemotactic protein-1” OR ”monocyte chemotactic protein 1” OR CysC OR ”cystatin C” OR CD4 OR biomarker* OR ((biological OR molecular) AND marker) OR prognostic OR prognosis OR predictive)

Domain 1 refers to: pediatric population

Domain 2 refers to: IgA vasculitis, with synonyms – Henoch Schonlein purpura, anaphylactoid purpura, allergic purpura

Domain 3 refers to: biomarkers that can predict the onset of renal involvement

Number of results: 4437

Notes: I used the truncations for words describing pediatric population to find all varieties of the words – neonate, neonates, neonatal, or pediatric, pediatrics and so on. I used the quotation mark for the biomarkers so I can find those exact biomarkers I am searching for.

Domain 3 subgroups:

**Hematological and inflammatory markers:** WBC OR "leukocyte count" OR NLR OR "neutrophil-to-lymphocyte ratio" OR "neutrophil to lymphocyte ratio" OR PLT OR MPV OR ”mean platelet volume” OR RDW OR ”red cell distribution width” OR ESR OR ”erythrocyte sedimentation rate” OR "C-reactive protein" OR CRP OR FIBRINOGEN OR Fib OR DD OR D-dimers OR ”serum sulfatide” OR ”erythrocyte glutathione S-transferase” OR e-GST

**Immunological tests:** IgA OR "immunoglobulin A" OR IgG OR "Immunoglobulin G" OR C3 OR C4 OR "Antiphospholipid antibodies" OR APLA OR APLAs OR "Anticardiolipin antibodies" OR "Anti-beta-2-glycoprotein-I antibod*" OR "anti-β2GPI antibod*" OR "Lupus anticoagulant" OR "lupus coagulation inhibitor"

**Renal damage specific markers:** "Apolipoprotein M" OR apoM OR ”Pentraxin 3” OR ”PTX 3” OR ”MMP-9” OR ”MMP 9” OR ”matrix metalloproteinase-9” OR ”TIMP-1” OR ”TIMP 1” OR ” tissue inhibitor matrix metalloproteinase-1” OR ”MMP-9/TIMP-1” OR ” MMP-9/TIMP-1 ratio” OR ACRU OR "albumin creatinine ratio" OR UACR OR KIM-1 OR ”KIM 1” OR ”kidney injury molecule – 1” OR ”kidney injury molecule 1” OR NAG OR ”N-acetyl-β-D-glucosamidase” OR NGAL OR ”Neutrophil gelatinase-associated lipocalin” OR MCP-1 OR ”MCP 1” OR ”monocyte chemotactic protein-1” OR ”monocyte chemotactic protein 1” OR CysC OR ”cystatin C” OR CD4

Database settings: I searched for All fields, with no other special settings and unchecked every checkmark below.

**CENTRAL (Cochrane)**

(neonat* OR pediatric* OR paediatric* OR adolescent OR adolescence OR adolescency OR child* OR young OR "young adult" OR "young adults" OR kids OR youth OR juvenile OR juvenility OR infant* OR infancy OR preschooler* OR teen OR teens OR teenager*)

AND

((Henoch AND (Schonlein OR Schönlein)) OR Henoch-Schonlein OR Henoch-Schönlein

OR HSP OR HSPN OR (IgA vasculitis) OR (IgA vasculitis nephritis) OR IgAV OR IgAVN OR (anaphylactoid AND purpura) OR (allergic AND purpura))

AND
("WBC" OR "leukocyte count" OR "NLR" OR "neutrophil-to-lymphocyte ratio" OR "neutrophil to lymphocyte ratio" OR "PLT" OR ”MPV” OR ”mean platelet volume” OR ”RDW” OR ”red cell distribution width” OR ”ESR” OR ”erythrocyte sedimentation rate” OR "C-reactive protein" OR "CRP" OR "FIBRINOGEN" OR "Fib" OR ”DD” OR ”D-dimers” OR ”serum sulfatide” OR ”erythrocyte glutathione S-transferase” OR e-GST OR "IgA" OR "immunoglobulin A" OR "IgG" OR "Immunoglobulin G" OR ”C3” OR ”C4” OR "Antiphospholipid antibodies" OR "APLA" OR "APLAs" OR "Anticardiolipin antibodies" OR (("Anti-beta-2-glycoprotein-I" OR "anti-β2GPI") NEXT antibod*) OR "Lupus anticoagulant" OR "lupus coagulation inhibitor" OR "Apolipoprotein M" OR "apoM" OR ”Pentraxin 3” OR ”PTX 3” OR ”MMP-9” OR ”MMP 9” OR ”matrix metalloproteinase-9” OR ”TIMP-1” OR ”TIMP 1” OR ” tissue inhibitor matrix metalloproteinase-1” OR ”MMP-9/TIMP-1” OR ” MMP-9/TIMP-1 ratio” OR "ACRU" OR "albumin creatinine ratio" OR "UACR" OR ”KIM-1” OR ”KIM 1” OR ”kidney injury molecule – 1” OR ”kidney injury molecule 1” OR ”NAG” OR ”N-acetyl-β-D-glucosamidase” OR ”NGAL” OR ”Neutrophil gelatinase-associated lipocalin” OR ”MCP-1” OR ”MCP 1” OR ”monocyte chemotactic protein-1” OR ”monocyte chemotactic protein 1” OR ”CysC” OR ”cystatin C” OR CD4 OR biomarker* OR ((biological OR molecular) AND marker) OR prognostic OR prognosis OR predictive)

Domain 1 refers to: pediatric population

Domain 2 refers to: IgA vasculitis, with synonyms – Henoch Schonlein purpura, anaphylactoid purpura, allergic purpura

Domain 3 refers to: biomarkers that can predict the onset of renal involvement

Number of results: 123

Notes: I used the truncations for words describing pediatric population to find all varieties of the words – neonate, neonates, neonatal, or pediatric, pediatrics and so on. I used the quotation mark for the biomarkers so I can find those exact biomarkers I am searching for.

Domain 3 subgroups:

**Hematological and inflammatory markers:** "WBC" OR "leukocyte count" OR "NLR" OR "neutrophil-to-lymphocyte ratio" OR "neutrophil to lymphocyte ratio" OR "PLT" OR ”MPV” OR ”mean platelet volume” OR ”RDW” OR ”red cell distribution width” OR ”ESR” OR ”erythrocyte sedimentation rate” OR "C-reactive protein" OR "CRP" OR "FIBRINOGEN" OR "Fib" OR ”DD” OR ”D-dimers” OR ”serum sulfatide” OR ”erythrocyte glutathione S-transferase” OR e-GST

**Immunological tests:** "IgA" OR "immunoglobulin A" OR "IgG" OR "Immunoglobulin G" OR ”C3” OR ”C4” OR "Antiphospholipid antibodies" OR "APLA" OR "APLAs" OR "Anticardiolipin antibodies" OR (("Anti-beta-2-glycoprotein-I" OR "anti-β2GPI") NEXT antibod*) OR "Lupus anticoagulant" OR "lupus coagulation inhibitor"

**Renal damage specific markers:** "Apolipoprotein M" OR "apoM" OR ”Pentraxin 3” OR ”PTX 3” OR ”MMP-9” OR ”MMP 9” OR ”matrix metalloproteinase-9” OR ”TIMP-1” OR ”TIMP 1” OR ” tissue inhibitor matrix metalloproteinase-1” OR ”MMP-9/TIMP-1” OR ” MMP-9/TIMP-1 ratio” OR "ACRU" OR "albumin creatinine ratio" OR "UACR" OR ”KIM-1” OR ”KIM 1” OR ”kidney injury molecule – 1” OR ”kidney injury molecule 1” OR ”NAG” OR ”N-acetyl-β-D-glucosamidase” OR ”NGAL” OR ”Neutrophil gelatinase-associated lipocalin” OR ”MCP-1” OR ”MCP 1” OR ”monocyte chemotactic protein-1” OR ”monocyte chemotactic protein 1” OR ”CysC” OR ”cystatin C” OR CD4

Database settings: I searched for clinical trials, with the option All text.

Table S1. Search key

Figure S1. Flowchart of study selection process

Table S2. Baseline characteristics of the included studies

| Study | Country | Study design | Study period | Patients’ age mean±SD | Patients’ age median (IQR) | Females percentage | Sample size | No. of patients with IgAVN | No. of patients with IgAV |
| --- | --- | --- | --- | --- | --- | --- | --- | --- | --- |
| AlKhater et al., 2020 | Saudi Arabia | retrospective cohort study | january 2009 - december 2018 | 7.1±3 | NA | NA | 113 | 31 | 82 |
| Al-Sheyyab et al., 1999 | Jordan | prospective case control study | may 1994 - april 1997 | 6.5±na | NA | 60.4 | 48 | 8 | 40 |
| Anil et al., 2009 | Turkey | retrospective cohort study | january 1996 - march 2006 | 7.9±2.9 | 8 | 47.7 | 430 | 192 | 238 |
| Ashari et al., 2024 | Iran | prospective cohort study | NA | 6.5±2.9 | NA | 25 | 47 | NA | NA |
| Avci et al., 2023 | Turkey | retrospective case control study | january 2011 - december 2020 | 7.9±3.2 | NA | 42.7 | 314 | 58 | 256 |
| Bi et al., 2022 (external validation cohort) | China | cross-sectional | june 2017 - august 2021 | NA | NA | 44.26 | 122 | 50 | 72 |
| Bi et al., 2022 (INTERNAL VALIDATION COHORT) | China | cross-sectional | august 2016 - april 2021 | NA | NA | 44.53 | 128 | 44 | 84 |
| Bi et al., 2022 (TRAINING SET) | China | cross-sectional | august 2016 - april 2021 | NA | NA | 44.92 | 138 | 48 | 90 |
| Brendel Muller et al., 2001 | Germany | retrospective cohort study | june 1997 - march 2001 | NA | NA | NA | 17 | NA | NA |
| Buscatti et al., 2018 | Brazil | retrospective cohort study | january 1983 - december 2015 | NA | NA | 50.33 | 296 | 139 | 157 |
| Cakici et al., 2019 | Turkey | retrospective cohort study | january 2001 - november 2016 | 7.4±3.43 | NA | 42.32 | 1120 | 414 | 706 |
| Cao et al., 2022 | China | prospective cohort study | october 2019 - december 2021 | 9.03±2.68 | NA | 47.5 | 240 | 153 | 87 |
| Carucci et al., 2022 | Italy | retrospective cohort study | may 2003 - june 2020 | NA | NA | 47.72 | 132 | 65 | 67 |
| Chaika et al., 2019 | Ukraine | retrospective cohort study | january 2015 - november 2018 | NA | NA | 40.96 | 83 | 25 | 58 |
| Chang H et al., 2015 | China | prospective case control study | october 2011 - november 2012 | 6±na | NA | 48.43 | 64 | 28 | 36 |
| Chang J et al., 2005 | China | retrospective cohort study | october 2002 - may 2003 | NA | NA | 35.41 | 48 | 21 | 27 |
| Chatpaitoon et al., 2024 | Thailand | bidirectional cohort study | january 2010 - december 2022 | NA | NA | 38.96 | 77 | 25 | 52 |
| Chen T et al., 2014 | China | retrospective cohort study | NA | NA | NA | NA | 72 | 31 | 41 |
| Chen X et al., 2008 | China | retrospective cohort study | NA | NA | NA | NA | 61 | 34 | 27 |
| Dalens et al., 1983 | France | prospective case control study | NA | NA | NA | NA | 45 | 6 | 39 |
| Danaci et al., 2024 | Turkey | prospective case control study | may 2014 - august 2015 | 9.21±4 | NA | 36.66 | 30 | 3 | 27 |
| Demir et al., 2021 | Turkey | retrospective cohort study | january 2006 - january 2016 | 8.49±3.35 | NA | 51.6 | 188 | 65 | 123 |
| Demir S et al., 2020 | Turkey | prospective case control study | NA | 8.42±3.56 | NA | 57.77 | 45 | 6 | 39 |
| Demircin et al., 1998 | Turkey | prospective case control study | september 1995 - march 1996 | 8.5±0.68 | NA | 56.2 | 16 | 6 | NA |
| Demircioglu Kilic et al., 2018 | Turkey | retrospective cohort study | october 2011 - december 2013 | 7.4±2.8 | NA | 47.8 | 186 | 28 | 158 |
| Ding et al., 2010 | China | prospective case control study | august 2006 - april 2008 | 8.2±na | NA | 34.6 | 78 | 40 | 38 |
| Dursun et al., 2011 | Turkey | prospective case control study | NA | NA | NA | 50 | 20 | 9 | 11 |
| Dyga K et al., 2020 | Poland | prospective case control study | 2016 - 2018 | NA | 7.5 (6-10) | 41.37 | 29 | 11 | 18 |
| Ekinci et al., 2019 | Turkey | retrospective cohort study | NA | 7.6±3.1 | NA | 43.5 | 214 | 16 | 198 |
| Elmas et al., 2014 | Turkey | retrospective cohort study | january 2006 - december 2010 | 7.56±3.3 | NA | 42.5 | 107 | 28 | 79 |
| Erol et al., 2016 | Turkey | prospective case control study | january 2014 - december 2015 | 7.65±3.41 | NA | 42.5 | 40 | 8 | 32 |
| Eryilmaz et al., 2016 | Turkey | retrospective cohort study | 1996 - 2002 | 9.2±2.7 | NA | 35.3 | 102 | 29 | 73 |
| Evans Jones et al., 1990 | UK | cross-sectional | NA | NA | NA | NA | 20 | NA | NA |
| Fan et al., 2017 | China | retrospective cohort study | january 2015 - december 2015 | 7.9±3 | NA | 32 | 50 | 17 | 33 |
| Fan et al., 2021 | China | retrospective cohort study | january 2015 - december 2019 | 8.3±3 | 8(6-10) | 41.3 | 2074 | 621 | 1453 |
| Fedakar et al., 2018 | Turkey | retrospective cohort study | april 1998 - june 2003 | NA | NA | NA | 80 | NA | NA |
| Fessatou et al., 2008 | Greece | prospective case control study | january 2005 - december 2006 | 6.3±3 | NA | 53.33 | 30 | 18 | 12 |
| Fretzayas et al., 2009 | Greece | retrospective cohort study | january 1992 - december 2000 | NA | 5.4 | 44.5 | 74 | 16 | 58 |
| Frkovic et al., 2024 | Croatia | prospective case control study | NA | NA | 6.83 | 36.08 | 97 | 42 | 55 |
| Fu et al., 2022 | China |  | january 2018 - december 2018 | NA | 7.2(5.64-9.52) | 42.5 | 1007 | 472 | 535 |
| Fuentes et al., 2014 | Mexico | prospective case control study | NA | NA | NA | 42.85 | 77 | 57 | 20 |
| Fujieda et al., 1998 | Japan | prospective case control study | NA | 8.3±na | NA | 44 | 25 | 15 | 10 |
| Ge et al., 2014 | China | prospective case control study | March 2010 - February 2013 | NA | NA | NA | 108 | 37 | 34 |
| Gokceoglu et al., 2020 | Turkey | retrospective cohort study | January 2013 - December 2019 | 8.4±3.8 | NA | 54.44 | 90 | 20 | 70 |
| Gungor et al., 2022 | Turkey | retrospective cohort study | February 2020 - October 2020 | 7.48±2.77 | NA | 46.7 | 75 | 8 | 40 |
| Ha et al., 2005 | Korea | prospective cohort study | NA | NA | NA | 50 | 28 | 18 | 10 |
| Han et al., 2023 | China | retrospective cohort study | january 2011 - june 2016 | NA | 8.5(6.92-10.67) | 47.67 | 451 | 387 | 64 |
| He et al., 2015 | China | prospective case control study | NA | NA | NA | NA | 12 | NA | NA |
| Held et al., 2024 | Croatia | prospective case control study | january 2020 - october 2023 | NA | 6.4(4.5-7.8) | 57 | 86 | 26 | 60 |
| Jauhola et al., 2010 | Finland | prospective cohort study | 1999 - 2006 | 7.1±3.5 | NA | 45.29 | 223 | 102 | 121 |
| Jia L et al., 2024 | China | prospective case control study | NA | 7.9±3.6 | NA | 64.63 | 82 | 21 | 61 |
| Jin Y et al., 2024 | China | prospective case control study | NA | NA | NA | NA | 16 | 8 | 8 |
| Jung et al., 2017 | Korea | retrospective cohort study | june 2003 - february 2016 | 7.62±3.76 | NA | 46.2 | 353 | 113 | 240 |
| Kaku et al., 1998 | Japan | retrospective cohort study | april 1988 - march 1995 | 6.3±2.7 | NA | 50 | 194 | 65 | 129 |
| Kamitsuji et al., 1987 | Japan | prospective case control study | NA | NA | NA | NA | 21 | NA | NA |
| Kandur et al., 2016 | Turkey | prospective case control study | september 2014 - september 2015 | NA | NA | NA | 36 | 9 | 27 |
| Kang et al., 2019 | China | prospective case control study | january 2018 - april 2018 | NA | NA | 41.6 | 125 | 67 | 58 |
| Kanik et al., 2015 | Turkey | prospective cohort study | september 2012 - january 2014 | 7.5±2.9 | NA | 56.06 | 66 | 19 | 47 |
| Karadag et al., 2021 | Turkey | prospective case control study | january 2016 - january 2019 | 7.5±3.5 | NA | 47.1 | 376 | 80 | 296 |
| Kim M et al., 2019 | Korea | retrospective cohort study | january 2007 - june 2018 | 6.1±0.7 | NA | 49 | 112 | 25 | 87 |
| Kim W et al., 2021 | Korea | retrospective cohort study | june 2005 - july 2020 | 6.6±2.9 | NA | 48.92 | 186 | 67 | 119 |
| Koc et al., 2020 | Turkey | retrospective cohort study | march 2008 - march 2012 | 8.1±3.1 | NA | 48.1 | 216 | 68 | 148 |
| Kocaoglu et al., 2017 | Turkey | retrospective cohort study | january 2012 - may 2015 | 7.7±3.4 | NA | 40 | 120 | 29 | 91 |
| Lau et al., 2007 | USA | prospective case control study | NA | 7.5±na | NA | NA | 18 | 12 | 6 |
| Lei et al., 2018 | China | retrospective cohort study | june 2012 - july 2014 | 8.3±2.4 | NA | 48.35 | 91 | 30 | 40 |
| Limpongsanurak et al., 2011 | Thailand | prospective cohort study | january 2003 - december 2007 | 6.9±2.6 | NA | 48.8 | 168 | 11 | 156 |
| Liu C et al., 2022 | China | retrospective cohort study | january 2020 - january 2021 | 8.4±2.98 | NA | 35.09 | 208 | 112 | 96 |
| Liu H et al., 2019 | China | prospective cohort study | january 2015 - december 2016 | 6.93±2.79 | NA | 36.8 | 163 | 60 | 103 |
| Liu L et al., 2023 | China | prospective case control study | june 2019 - june 2020 | NA | NA | 51.08 | 92 | 30 | 62 |
| Liu Y et al., 2022 | China | prospective case control study | november 2018 - may 2019 | 9.17±3.08 | NA | 51.7 | 58 | 13 | 45 |
| Ma D et al., 2017 | China | prospective case control study | january 2015 - december 2016 | 7.81±2.63 | NA | 42.05 | 264 | 103 | 147 |
| Ma X et al., 2022 | China | retrospective cohort study | january 2019 - october 2021 | NA | NA | 40 | 125 | 27 | 47 |
| Ma Y et al., 2020 | China | prospective case control study | NA | NA | NA | 45.23 | 42 | 14 | 28 |
| Makieieva et al., 2019 | Ukraine | prospective case control study | january 2015 - november 2018 | NA | NA | 40.96 | 83 | 25 | 58 |
| Makieieva et al., 2020 | Ukraine | prospective case control study | NA | NA | NA | NA | 60 | 20 | 40 |
| Mao Y et al., 2012 | China | prospective cohort study | june 2007 - june 2010 | 6.68±2.41 | NA | 53.27 | 107 | 56 | 51 |
| Mao Y et al., 2014 | China | retrospective cohort study | january 2006 - june 2012 | 6.9±2.7 | NA | 42.42 | 535 | 267 | 268 |
| Marro et al., 2022 | UK | prospective case control study | august 2019 - september 2021 | 8.2±4.1 | NA | 33.89 | 59 | 12 | 35 |
| Marro et al., 2022 | UK | cross-sectional | august 2019 - october 2021 | NA | NA | 50 | 8 | 4 | 4 |
| Marro et al., 2023 | UK | cross-sectional | august 2019 - may 2022 | NA | NA | 48.57 | 35 | 15 | 20 |
| Min et al., 2024 | China | retrospective cohort study | june 2018 - december 2020 | NA | NA | 49.57 | 117 | 68 | 49 |
| Muller et al., 2000 | Germany | prospective cohort study | 1997 - 1998 | NA | 5.3 | 41.66 | 36 | 15 | 21 |
| Muslu et al., 2002 | Turkey | prospective case control study | january 2000 - april 2001 | 9.7±3.4 | NA | 43.33 | 30 | NA | NA |
| Onder et al., 2022 | Turkey |  | december 2010 - december 2020 | 14.9±4.1 | NA | 41.05 | 95 | 57 | 38 |
| Ozel et al., 2018 | Turkey | retrospective cohort study | january 2013 - december 2016 | 7.82±3.01 | NA | 36.97 | 119 | 35 | 84 |
| Pan Y et al., 2014 | China | prospective case control study | january 2010 - december 2013 | 5.24±na | NA | 43.18 | 1232 | NA | NA |
| Pillebout et al., 2017 | France | prospective case control study | april 2010 - may 2015 | NA | 7(6-9) | 44 | 50 | 33 | 17 |
| Qin et al., 2011 | China | prospective case control study | march 2006 - march 2009 | NA | NA | NA | 134 | 66 | 68 |
| Qin W et al., 2010 | China | prospective case control study | NA | 7.4±1.9 | NA | 34.3 | 32 | 15 | 17 |
| Rigante et al., 2005 | Italy | prospective cohort study | 1992 - 2002 | 6.33±2.54 | NA | 50 | 74 | 14 | 60 |
| Ru et al., 2013 | China | prospective case control study | july 2009 - december 2011 | NA | NA | 45.76 | 59 | 28 | 31 |
| Ruan J et al., 2022 | China | prospective cohort study | january 2015 - january 2021 | 7.9±2.97 | 8 | 43 | 1683 | 448 | 1235 |
| Sano et al., 2002 | Japan | retrospective cohort study | january 1991 - december 2000 | 6.3±2.4 | NA | 37 | 134 | 65 | 69 |
| Saulsbury et al., 1998 | USA | prospective case control study | NA | 6.3±2.6 | NA | 51.28 | 19 | NA | 20 |
| Sestan et al., 2021 | Croatia | retrospective cohort study | 2009 - 2019 | NA | 6.33 | 47.62 | 611 | 130 | 481 |
| Shen et al., 2022 | China | cross-sectional | january 2016 - october 2019 | 10.97±3.56 | NA | 44.73 | 351 | 75 | 276 |
| Shin et al., 2006 | Korea | retrospective cohort study | 1996 - 2001 | 7.2±2.8 | NA | 45.14 | 206 | 78 | 128 |
| Simila et al., 1977 | Finland | prospective cohort study | 1973 - 1975 | 7±3.1 | NA | 42.3 | 26 | 5 | 10 |
| Song et al., 2022 | China | retrospective cohort study | january 2016 - december 2019 | NA | NA | NA | 261 | NA | NA |
| Su et al., 2016 | China | prospective case control study | NA | 6.76±2.04 | NA | 39 | 92 | 36 | 56 |
| Sun et al., 2017 | China | prospective case control study | february 2013 - january 2014 | 8.74±2.99 | NA | 35.77 | 109 | 57 | 52 |
| Sunar et al., 2023 | Turkey | retrospective cohort study | january 2005 - july 2020 | 7.74±3.36 | NA | 44.71 | 539 | 105 | 434 |
| Tanaka H et al., 2023 | Japan | retrospective cohort study | october 2013 - september 2022 | NA | 6.4 | 36.36 | 22 | NA | NA |
| Tang et al., 2021 | China | retrospective cohort study | june 2016 - june 2018 | NA | NA | 54.21 | 83 | 57 | 26 |
| Ture et al., 2021 | Turkey | prospective case control study | january 2018 - january 2019 | 10.01±3.26 | NA | 55.7 | 61 | 23 | 38 |
| Wang B et al., 2020 | China | prospective case control study | april 2018 - january 2019 | NA | NA | 55.5 | 36 | 11 | 25 |
| Wang J et al., 2017 | China | prospective case control study | january 2013 - december 2015 | 8.81±3.6 | NA | 46.4 | 345 | 126 | 135 |
| Wang J et al., 2017 | China | prospective case control study | october 2014 - october 2015 | 8±3.12 | NA | 52.6 | 76 | 35 | 41 |
| Wang J et al., 2022 | China | prospective cohort study | november 2018 - october 2021 | NA | NA | NA | 288 | 114 | 174 |
| Wang J et al., 2023 | China | retrospective cohort study | february 2017 - october 2019 | 7.23±2.63 | NA | 47.3 | 605 | 110 | 495 |
| Wright et al., 2022 | UK | cross-sectional | NA | NA | NA | NA | 47 | 10 | 37 |
| Wu et al., 2019 | China | prospective case control study | march 2017 - december 2017 | 9.04±0.71 | NA | 39.44 | 109 | 43 | 66 |
| Xi et al., 2021 | China | retrospective cohort study | june 2019 - december 2020 | NA | NA | 52.05 | 146 | 50 | 96 |
| Xu et al., 2017 | China | retrospective cohort study | june 2012 - may 2015 | 7.6±2.8 | NA | 44.6 | 669 | 256 | 413 |
| Yakut et al., 2020 | Turkey | retrospective cohort study | january 2015 - 2018 | 8.7±3.6 | NA | 42.25 | 71 | NA | NA |
| Yeganeh et al., 2024 | Iran | cross-sectional | september 2015 - june 2017 | 6.29±1.35 | NA | 35.4 | 48 | 14 | 34 |
| Yildiz B et al., 2008 | Turkey | prospective case control study | january 2004 - may 2007 | 8.6±3.9 | NA | 31.81 | 44 | 20 | 24 |
| Yoshioka et al., 1980 | Japan | prospective cohort study | NA | NA | NA | NA | 31 | 20 | 11 |
| Yu et al., 2024 | China | prospective case control study | november 2022 - april 2023 | NA | NA | 43 | 60 | 30 | 30 |
| Yu S et al., 2024 | China | prospective cohort study | may 2023 - august 2023 | NA | NA | 49.12 | 57 | 17 | 17 |
| Yuan et al., 2018 | China | prospective case control study | june 2016 - june 2017 | NA | NA | 47.32 | 112 | 54 | 58 |
| Yuksel et al., 2015 | Turkey | prospective cohort study | february 2011 - october 2012 | 8.01±2.52 | NA | 45 | 60 | 29 | 31,00 |
| Zhang J et al., 2015 | China | prospective case control study | january 2013 - december 2013 | NA | NA | 37.28 | 59 | 32 | 27 |
| Zhang N et al., 2017 | China | retrospective cohort study | january 2013 - october 2015 | NA | 10 | 45.21 | 502 | NA | NA |
| Zhang Y et al., 2017 | China | prospective case control study | june 2013 - january 2015 | 6.3±2.8 | NA | 41.3 | 92 | 31 | 61 |
| Zhang Y et al., 2022 | China | retrospective cohort study | june 2021 - june 2022 | 8.3±0.1 | NA | 47.12 | 174 | 45 | 129 |
| Zhao et al., 2019 | China | retrospective cohort study | january 2016 - december 2017 | 7.84±2.81 | NA | 48.14 | 135 | 15 | 120 |
| Zhu L et al., 2022 | China | prospective case control study | january 2017 - december 2018 | 6.58±2.84 | NA | 40.38 | 260 | 27 | 233 |
| Zhu X et al., 2017 | China | prospective cohort study | january 2013 - december 2015 | NA | NA | 28 | 225 | 120 | 105 |
| Zhu X et al., 2023 | China | retrospective cohort study | january 2017 - december 2022 | 10.5±na | NA | NA | 79 | 22 | 57 |
| Zhu Z et al., 2023 | China | prospective cohort study | 2020 - 2022 | NA | NA | NA | 28 | 18 | 10 |

Table S3. Main findings of the studies included in the Systematic Review

| Study | IgAVN definition | Biomarkers analysed | Results |
| --- | --- | --- | --- |
| AlKhater et al., 2020 | hematuria - >5rbc/hpf or dipstick + or gross hematuria, proteinuria - dipstick + or urine spot protein/creatinine ratio >0.2 or >4mg/m2/24h; high BP - average systolic/diastolic BP>95th percentile, AKI - KDIGO criteria | Hb, ESR, PLT, WBC, CRP, ASOT | no statistically significant difference between patients with and without IgAVN |
| Al-Sheyyab et al., 1999 | proteinuria and/or hematuria | fecal calprotectin | no statistically significant difference between patients with and without IgAVN |
| Anil et al., 2009 | NA | ASOT | no statistically significant difference between patients with and without IgAVN |
| Ashari et al., 2024 | proteinuria, hematuria, decreased GFR <90ml/min | XGBoost model; significant correlation - WBC, Ne, Eo, Alb, SCr, IgG, IgA, IgE, uRBP, uNAG, 24h-urinary prot | training set AUC:0.895 (0.827-0.963); test set AUC:0.87 (0.799-0.941) |
| Avci et al., 2023 | hematuria - >5rbc/hpf or positive dipstick, proteinuria - positive dipstick or protein/creatinine ratio >20mg/mmol, AKI according to KDIGO criteria | WBC, PLT, CRP, ESR, ASOT, C3, C4, Albumin | increased CRP and decreased albumin in patient with IgAVN (p=0.0001) |
| Bi et al., 2022 | NA | WBC, Ne, Ly, Mo, PLT, RBC, Hb, MCV, RDW, PDW, MPV, CRP, D-dimers, APTT, Fib, IgA, IgM, IgG, C3, C4 | lower PLT levels in IgAVN (p = 0.019 and p=0.0001), higher MCV levels in IgAVN (p=0.0001), higher RDW levels in IgAVN (p=0.001), lower CRP levels in IgAVN (p=0.0001), lower D-dimers levels in IgAVN (p=0.0001), lower IgA levels in IgAVN (p=0.022), lower IgG levels in IgAVN (p=0.0001), higher IgM levels in IgAVN (p=0.001) |
| Brendel Muller et al., 2001 | hematuria and or proteinuria | fibrinogen, d-dimers, fXIIII activity | small sample size, no difference between patients with and without IgAVN |
| Buscatti et al., 2018 | hematuria - >5rbc/hpf or red cells casts, proteinuria - >0.1g/m2/24h, high blood pressure - systolic and/or diastolic pressure > 95th percentile on >3 occasions, AKI - serum creatinine >2mg/dl or based on modified IgAVNFLE criteria | serum IgA | no association between increased serum IgA and IgAVN |
| Cakici et al., 2019 | hematuria - > 5rbc/hpf, proteinuria - positive dipstick test or >4mg/m2/24h, HBP - average systolic or diastolic BP > 95th percentile, AKI - oliguria and/or azotemia | ESR, CRP | increased CRP and ESR in IgAVN patients (p<0.001); ESR>20mm/h - risk factor for nephritis in both univariate and multivariate analysis (p<0.001) |
| Cao et al., 2022 | NA | IFNg, IL-4, IFNg/IL-4, IL-6, TLR2, TLR4 | TLR2, TLR4 mRNAhigher in IgAVN |
| Carucci et al., 2022 | hematuria - gross hematuria or >5 rbc/hpf, proteinuria - >4mg/m2/24h or >5mg/kg/24h or proteinuria/uriNAry creatinine >0.2 mg/mg, AKI - increase in serum creatinine >=1.5 times baseline or urine output < 0.5ml/kg/h for 6h | WBC, Ne, Ly, NLR, PLR, PLT, CRP, ESR, IgA | no statistically significant difference between patients with and without IgAVN |
| Chaika et al., 2019 | hematuria - >5cells/mm3, proteinuria | WBC, ESR, Fibrinogen, PLT, IL-8, TNFa | ESR is decreased in patients with IgAVN (p=0.09) |
| Chang H et al., 2015 | NA | fXIII activity, fibrinogen | fXIII lower in patients with systemic involvement |
| Chang J et al., 2005 | NA | NO, ET-1, NO/ET-1 ratio | NO, ET-1, NO/ET-1 ratio are increased in IgAVN patients (p<0.01) |
| Chatpaitoon et al., 2024 | hematuria - >5rbc/hpf or red cells casts, proteinuria - positive dipstick or >0.3g/24 or UPCR>0.3 g/g; AKI and CKD based on KDIGO criteria | Hb, WBC, Ne, PLT, ESR, CRP, Alb, C3, ASOT, ANA | no statistically significant difference between patients with and without IgAVN |
| Chen T et al., 2014 | hematuria and or proteinuria | CysC, NGAL | small sample size, promising biomarkers for early diagnosis of IgAVN (NGAL - AUC= 0.789, CysC - AUC = 0.692) |
| Chen X et al., 2008 | NA | serum and urinary LTB4 and urinary LTE4 | serum and urinary LTB4 and urinary LTE4 are increased in IgAVN patients (p<0.01) |
| Dalens et al., 1983 | NA | urinary hepatocyte growth factor and kidney staining for HGF | higher in patients with microalbuminuria, lower in patients with macroalbuminuria (both uHGF and kidney expression) p<0.01 |
| Danaci et al., 2024 | NA | MDA, SOD, pGSN | no statistically significant difference between patients with and without IgAVN |
| Demir et al., 2021 | hematuria - >5rbc/hpf or red cells casts, proteinuria > 4mg/m2/24h, high blood pressure - systolic and/or diastolic pressure > 95th percentile on >3 occasions, AKI | Hb, WBC, PLT, IgA, IgG, IgM, C3, C4, ESR | higher levels of ESR in IgAVN patients (p=0.048) |
| Demir S et al., 2020 | NA | WBC, PLT, CRP, metabolite profiling | no statistically significant difference between patients with and without IgAVN for WBC, PLT, CRP; DHAP, prostaglandin D2/I2, porphobilinogen, 5-methyltetrahydrofolic acid, N-acetyl-4-O-acetylneuraminic acid/ N-acetyl-7-O-acetylneuraminic acid may serve as biomarkers for predicting IgAVN |
| Demircin et al., 1998 | NA | serum superoxid dismutase activity, serum malondialdehyde | no statistically significant difference between patients with and without IgAVN |
| Demircioglu Kilic et al., 2018 | hematuria and or proteinuria | CRP | high CRP level associated with IgAVN (p=0.007) |
| Ding et al., 2010 | hematuria >5rbc/hpf, red blood cells casts, >2+ dipstick, proteinuria - >300mg/24h,UACR >30mmol/mg | ASOT | no statistically significant difference between patients with and without IgAVN |
| Dursun et al., 2011 | hematuria - macroscopic or microscopic >5rbc/hpf, proteinuria - positive dipstick test or >4mg/m2/24h | Hb, WBC, PLT, ESR, CRP, Eo, IgA, IgE, C3, C4, CD144+EMP/microliter, CD146+EMP/microliter | no statistically significant difference between patients with and without IgAVN |
| Dyga K et al., 2020 | hematuria - >5rbc/hpf, proteinuria > 30mg/mmol (UPCR), eGFR < 60ml/min/m2 | Hb, WBC, PLT, CRP, ASOT, C3, C4, D-dimers, IgA, serum and urinary NGAL, L-FABP, KIM-1 | no statistically significant difference between patients with and without IgAVN |
| Ekinci et al., 2019 | biopsy proven IgAVN | PLT, MPV, Ne, Ly, NLR, PLR | Ne count is significantly higher in patients with IgAVN than in those without IgAVN (p=0.004) |
| Elmas et al., 2014 | hematuria and or proteinuria, high BP - systolic or diastolic average >95th percentile | Hb, WBC, PLT, ESR, CRP, IgA, IgG, IgM, C3, C4 | PLT count is predictive for IgAVN (OR=1, AUC=0.66, p=0.009) |
| Erol et al., 2016 | hematuria - >5rbc/hpf, proteinuria > 0.5g/L | serum and urine MMP-9, | the urinary MMP-9 level of IgAVN patients is significantly higher than in patients with IgAV (p<0.005) |
| Eryilmaz et al., 2016 | NA | C3, ESR | Elevated C3 and ESR are associated with IgAVN (p=0.033, p=0.003) |
| Evans Jones et al., 1990 | NA | PLT | no statistically significant difference between patients with and without IgAVN |
| Fan et al., 2017 | hematuria and or proteinuria | vitamin D levels | the deficiency and severe deficiency of vitamin D was associated with IgAVN (p<0.05) |
| Fan et al., 2021 | hematuria >5rbc/hpf and proteinuria >1g/24h and/or hypertension (>95th percentile) and/or elevated plasma level of blood urea nitrogen and creatinine > 100% of upper limit of normal range | IgG, IgA IgM, C3,C4 | no statistically significant difference between patients with and without IgAVN |
| Fedakar et al., 2018 | hematuria, proteinuria, AKI, high BP, decreased urine density | Hb, WBC, MCV, PLT | no statistically significant difference between patients with and without IgAVN |
| Fessatou et al., 2008 | hematuria - >3-5rbc/hpf, proteinuria > 30mg/dl or microalbuminuria | urinary and plasma ET-1 | no statistically significant difference between patients with and without IgAVN |
| Fretzayas et al., 2009 | hematuria >10rbc/hpf or proteinuria >30mg/dl, that persists >7days | anti endothelial cells antibodies IgG and IgA, thrombomodulin | association between AECA IgA and thrombomodulin and proteinuria over 1g/day (p<0.01) |
| Frkovic et al., 2024 | hematuria - >5rbc/hpf or proteinuria - >0.15g protein/dU | ESR, CRP, Hb, Ly, PLT, Ferritin, IgG, IgM, IgA, e-GST activity | IgA, IgM significantly higher in the IgAVN group (p<0.05); e-GST activity is higher in patients with IgAVN (p<0.001) |
| Fu et al., 2022 | hematuria - >5rbc/hpf or red blood cells casts or >+2 on dipstick test, proteinuria - >0.3g/24h or >30mmol/mg of urine albumine/creatinine ratio | WBC, Hb, PLT,RDW-CV, CRP, ESR, C3, C4, IgG, IgA, IgM, IgE, TG, Chol, low density lp, albumin | increased RDW-CV, decreased C3, decreased IgG, elevated TG are independent influencing factors of IgAVN |
| Fuentes et al., 2014 | hematuria - macroscopic or microscopic >5rbc/hpf, proteinuria - >4mg/m2/24h | urinary MCP-1/creatinine | urinary MCP-1/creatinine levels are significantly increased in the IgAVN group compared with IgAV (p=0.0001) |
| Fujieda et al., 1998 | hematuria and or proteinuria | antiphospholipid antibodies | moderate correlation between aPL antibodies and IgAVN |
| Ge et al., 2014 | hematuria and or proteinuria | urinary microalbumin, urinary beta-2-microglobulin, serum PTX3, CRP | plasma PTX 3 is higher in the IgAVN group compared with IgAV (p<0.05) |
| Gokceoglu et al., 2020 | elevated kidney funtion tests, hematuria > 5rbc/hpf, proteinuria - >4mg/m2/h or > 0.2mg/mg UPCR | WBC, Ne, Ly, PLT, NLR, RDW, MPV, CRP, ESR | mean neutrophil count, RDW, CRP are higher in patients with IgAVN |
| Gungor et al., 2022 | hematuria - >5rbc/hpf, proteinuria > 30mg/dl in spot urine, UPC > 0.5 in patients below 2y, > 0.2 in patients above 2y | immature granulocytes percentage, WBC, Ne, PLT, NLR, PLR, MPV, MPR, CRP, ESR, systemic immune-inflammation index | immature granulocytes percentage, WBC, Ne, PLT, NLR, PLR, MPV, MPR, systemic immune-inflammation index are statistically higher in patients with systemic organ involvement (p < 0.05), but there is no statistically significant difference between IgAVN and gastrointestinal involvement |
| Ha et al., 2005 | NA | TNF alpha, albumin | serum TNFa are significantly higher in patients with IgAVN with proteinuria compared to IgAVN without proteinuria and IgAV in the acute phase (p<0.005) |
| Han et al., 2023 | hematuria - macroscopic or >3rbc/hpf 3 times within 1 week, proteinuria - 3 times within 1 week - >150mg/24h, UPCR>0.2, urine microalbumin is elevated | uric acid, cystatin c | Significant difference between uric acid in patients with and without IgAVN (p<0.05) |
| He et al., 2015 | hematuria > 5rbc/hpf, proteinuria >300mg/24h | IgA, C3, C4, CRP, proteomics analysis - serum samples | IgAVN patients exhibited higher serum angiotensinogen levels than IgAV patients (p=0.0005), and lower serum amyloid A levels (p=0.031) |
| Held et al., 2024 | NA | serum and urine Gd-IgA1, HMGB1, RAGE, PCDH1 | no statistically significant difference between patients with and without IgAVN |
| Jauhola et al., 2010 | hematuria > 5 rbc/hpf or dipstick test +/++ in 3 consecutive days, +++ in 2 consecutive days, proteinuria - >200mg/l, urine albumin > 30mg/l or positive dipstick test, or nephrotic-nephritic syndrome | ESR, CRP, IgA, IgE, IgG, IgM, C3, C4, ANCA | no statistically significant difference between patients with and without IgAVN |
| Jia L et al., 2024 | NA | 440-plexed protein array screen | ADAM12, CEACAM-5 - positively correlated with the severity of IgAVN |
| Jin Y et al., 2024 | hematuria and or proteinuria | f XIII activity | decreased fXIII activity is a risk factor for IgAVN |
| Jung et al., 2017 | hematuria and or proteinuria | WBC, PLT, IgA, uric acid, Hb, CRP, ASOT | high uric acid is statistically associated with IgAVN (p=0.034) |
| Kaku et al., 1998 | hematuria and or proteinuria | plasma gelsolin | higher level of pGSN may be a risk factor for IgAVN |
| Kamitsuji et al., 1987 | NA | f XIII activity, fibrinogen, FDP, f VIII activity | no statistically significant difference between patients with and without IgAVN |
| Kandur et al., 2016 | hematuria and or proteinuria | WBC, PLT, MPV, PDW, CRP, ESR | higher levels in patients with IgAV, but no difference between the systemic involvements; MPV - lower |
| Kang et al., 2019 | hematuria and or proteinuria | IgA, IgG, IgM, C3, C4, serum Gd-IgA1, urine Gd-IgA1/creatinine ratio | serum Gd-IgA1, urine Gd-IgA1/creatinine ratio are significantly higher in patients with IgAVN (p<0.05) |
| Kanik et al., 2015 | NA | fecal calprotectin | fecal calprotectin is sigificantly higher in patients with IgAVN (p=0.017) |
| Karadag et al., 2021 | hematuria - >5rbc/hpf, rbc casts, proteinuria - >4mg/m2/h, hypertension - BP>95th percentile, kidney insufficiency | WBC, Hb, PLT, Ne, Ly, NLR, PLR, MPV, MPR, CRP, ESR | NLR and PLR are significantly higher in patients with IgAVN (p=0.01; p=0.08) |
| Kim M et al., 2019 | hematuria - > 5rbc/hpf, proteinuria - >0.15g/day, or >4mg/m2/h | WBC, Ne, Ly, Hb, MCV, RDW-SD, PLT, MPV, ESR, CRP, NLR, PLR | Ne, RDW-SD, NLR are significantly higher in patients with IgAVN (p=0.011, p=0.032, p=0.027) |
| Kim W et al., 2021 | hematuria - >5rbc/hpf or >+2 dipstick, proteinuria spot urine >30mg/dl, UPCR > 0.5 in children <2y, and >0.2 in children >2y | WBC, Ne, Ly, NLR, PLT, PLR, MPV, MPR, Hb, RDW-CV, CRP | Ly are significantly lower in IgAVN patients (p=0.001), NLR and PLR are significantly higher in patients with IgAVN (p=0.002) |
| Koc et al., 2020 | microscopic or macroscopic hematuria, proteinuria - >4mg/m2/h | Hb, Le, PLT, IgA, IgG, IgM, IgE, ESR, CRP, ASOT, C3, C4, Fib, Albumin | Hb is significantly highe in patients with IgAVN (p=0.04) |
| Kocaoglu et al., 2017 | hematuria and or proteinuria | Helix aspersa (HAA)-binding IgA1 | slight elevation in patients with IgAVN compared to IgAV, but not statistically significant |
| Lau et al., 2007 | hematuria and or proteinuria | WBC, Ne, Ly, PLT, PLT, MPV, CRP and previous infections serology | lower MPV levels may predict IgAVN in Mycoplasma pneumonia triggered IgAV |
| Lei et al., 2018 | NA | IgG, IgM, IgA, IgE, C3, CD4+ T cells, CD8+ T cells, CD4+/CD8+ ratio | IgA and IgG are higher in the IgAVN group (p<0.01) |
| Limpongsanurak et al., 2011 | NA | ASOT | no statistically significant difference between patients with and without IgAVN |
| Liu C et al., 2022 | biopsy proven IgAVN | lipidomics analysis | phosphatidyl coline decrease - prominent biomarker for IgAVN |
| Liu H et al., 2019 | NA | urinary NAG, B2MG, RBP, TRF, IgG, MA | uIgG (OR 1.48, 95%95%CI 1.23-1.85), log(uMA) (OR 2.31, 95%95%CI 1.76-3.21), uTRF (OR 1.5, 95%95%CI 1.17-2.22) were higher in the IgAVN group |
| Liu L et al., 2023 | NA | WBC, PLT, MPV, NLR, PLR, Eo, IgA, IgE, IgG, IgM, C3, C4, D-dimers, serum B2MG, urinary B2MG, albumin, cystatin C, FOBT and serum proteomic profiles | serum IgA level is significantly higher in patients with IgAVN (p=0.002); the proteomic profiles only outlined candidate proteins |
| Liu Y et al., 2022 | hematuria and or proteinuria | MCP-1, NO2, NO3, S-nitrosotiol | good prognostic value in predicting IgAVN |
| Ma D et al., 2017 | NA | WBC, PLT, Chol, LDL | WBC, Chol and LDL are significantly higher in patients with IgAVN (p=0.00, p=0.023, p=0.00); PLT are lower in patients with IgAVN (p=0.000) |
| Ma X et al., 2022 | hematuria > 5 rbc/field, proteinuria ?300mg/24h | Ne, Ly, total proteins, albumin, globulin, transthyretin, NLR, CD3-HLADR+ B lymphocyte counts, CD19+ B lymphocyte counts | total protein (p=0.008), albumin (p=0.001), transthyretin (p<0.001) were significantly lower in patients with IgAVN, CD3-HLADR+ B lymphocyte counts, CD19+ B lymphocyte counts were higher in the IgAVN group (p<0.05) |
| Ma Y et al., 2020 | Chinese Society Evidence-Based Guidelines criteria for nephritis | UAGT(angiotensinogen)/Ucr, urinary fibroblast specific protein 1, urinary thrombin | UAGT(angiotensinogen)/Ucr, urinary fibroblast specific protein 1, urinary thrombin are all increased in IgAVN patients |
| Makieieva et al., 2019 |  |  |  |
| Makieieva et al., 2020 | hematuria and or proteinuria | urinary angiotensinogen/urinary creatinine ratio | u ang/u cr ratio correlates with serum creatinine and 24h urinary protein excretion in IgAVN, potential biomarker for severity of IgAVN (higher in patients with IgAVN with proteinuria compared with IgAVN with hematuria) |
| Mao Y et al., 2012 | UACR over 30mg/mmol | urinary and plasma CfD, CfB, Masp-1 | urinary concentrations significantly higher in IgAVN for CfB and CfD and non significant differences in plasma + urinary Masp-1 |
| Mao Y et al., 2014 | hematuria >5rbc/hpf, proteinuria >150mg/24h, at 2 different time points within 6 months of IgAV onset | FOBT, WBC, Ne, PLT, MPV, CRP, Albumin, Globulin, D-dimers, Fibrinogen, CysC, CD4+ T cells, CD8+ T cells, CD4/CD8 ratio, CD19+ B cells, NK cells, IgA, IgM, IgG | positive FOBT is an independent risk factor for IgAVN (adjusted OR=2.14, 95%95%CI = 1.35 - 3.37) |
| Marro et al., 2022 | UACR>30mg/mmol | serum and urinary IgA | urinary IgA can distinguish between patients with and without IgAVN (AUC=0.838, 95%CI=0.704 - 0.973, p<0.01) |
| Marro et al., 2022 | hematuria and or proteinuria | urinary alpha1 microglobulin, NAG | correlation between the tubular markers and development of nephritis and IgAVN |
| Marro et al., 2023 | UACR over 30mg/mmol and biopsy proven IgAVN | human kidney biomarker array, human XL cytokine array | urinary protein profiles do vary between IgAV and IgAVN, but due to the small sample size and semiquantitative technique used |
| Min et al., 2024 | hematuria and or proteinuria >150mg/24h | ESR, CRP, IgG, IgA, IgM, D-dimers, WBC, Albumin, PLT, APTT | a D-dim level over 1,62 mg/L is associated with the risk of IgAVN (adjusted OR=1.259, 95%CI = 1.02-1.55) |
| Muller et al., 2000 | hematuria and or proteinuria | Ils 2,4,6,10, TNFa, IFNg, Igs A, E, M, G, C3, C4, CRP, CD3, CD4, CD8, CD 20, CD3-CD16+CD56+ | higher levels of Ig and lower levels of complement in patients with IgAVN; correlation between proteinuria and IgE levels |
| Muslu et al., 2002 | NA | WBC, ESR, CRP, ASOT, IgA, ET-1 | no statistically significant difference between patients with and without IgAVN |
| Onder et al., 2022 | hematuria > 5rbc/hpf, proteinuria UPCR>0.5mg/mg in children aged <2y, UPCR>0.2 mg/mg in children aged>2y | Hb, Le, Ne, Ly, Mo, PLT, MPV, PCT, PDW, PLR, NLR, RDW, IgA, C3, ASOT | Ne (p=0.011), PDW(p=0.028), PLR (p=0.015), NLR(p<0.01) are significantly higher in patients with IgAVN, while Ly (p=0.003) are significantly lower in patients with IgAVN |
| Ozel et al., 2018 | macroscopic or microscopic hematuria (>5rbc/hpf) AND proteinuria | PLT, MPV, Ne, Ly, NLR | no statistically significant difference between patients with and without IgAVN |
| Pan Y et al., 2014 | hematuria and or proteinuria | OX40 expression on CD4 and CD8 + T cells and serum OX40L level | IgAV is associated with an upregulation of OX40/OX40L system |
| Pillebout et al., 2017 | hematuria - positive dipstick or >5 rbc/hpf, proteinuria - UPCR >0.5 g/g, eGFR<60ml/min | serum and urinary IgA, IgM, IgG, IgD, IgE, IL-1B, IL-6, IL-8, IL-10, IL-12p70, TNFa, NGAL | serum Gd-IgA1, urine IgA (p<0.0001), IgG (p<0.01), IgM (p<0.0001), IL-6 (p<0.01), IL-8(p<0.01), IL-10(p<0.01) are accurate predictors of IgAVN |
| Qin et al., 2011 | hematuria - >5rbc/hpf, proteinuria > 0.5g/L | serum and urine MMP-9, TIMP-1, MMP-9/TIMP-1 ratio | serum and urine MMP-9, TIMP-1, MMP-9/TIMP-1 ratio are significantly increased in IgAVN patients (p<0.05) |
| Qin W et al., 2010 | hematuria and or proteinuria | WBC, Ne, Ly, Eo, RBC, PLT, Hb, IgA, IgG, IgM, IgE, C3, C4 | no statistically significant difference between patients with and without IgAVN |
| Rigante et al., 2005 | hematuria > 5rbc/hpf, proteinuria >30mg/dl, >150mg/24h | ESR,CRP, WBC, IgA, C3, C4 | no statistically significant difference between patients with and without IgAVN |
| Ru et al., 2013 | NA | IGF-1, IGFBP-3, IgG, IgM, IgA, C3, IgA/C3 ratio, WBC, RBC, Hb, PLT | IGF-1 (p<0.05), IGFBP-3 (P<0.05) are significantly higher in patients with IgAVN |
| Ruan J et al., 2022 | hematuria and or proteinuria | f XIII activity and serum IgA levels | fXIII significantly decreased in patients with IgAVN |
| Sano et al., 2002 | hematuria and or proteinuria | f XIII activity | correlation between decreased factor XIII activity and prolonged hospitalization and increased CS use |
| Saulsbury et al., 1998 | hematuria > 5 rbc/hpf, proteinuria >30mg/dl | IgD | IgD levels are lower in IgAVN patients (p=0.02) |
| Sestan et al., 2021 | hematuria >5rbc/hpf or >+2 dipstick or red cells casts, proteinuria >0.3g/24h or UACR > 30mmol/mg | CRP, ESR, APTT | elevated ESR and CRP, as well as APTT are associated with a higher risk of IgAVN (p<0.03) |
| Shen et al., 2022 | NA | IgG, IgA, IgM, C3, C4, WBC, RBC, PLT, MPV, RDW, NLR, PLR, ALB, CysC | IgG and Alb were significantly lower in patients with IgAVN (p<0.003) |
| Shin et al., 2006 | hematuria - macroscopic or microscopic >5rbc/hpf, proteinuria - significant proteinuria > 20mg/m2/h | WBC, PLT, CRP, ASOT, IgA, C3 | no statistically significant difference between patients with and without IgAVN |
| Simila et al., 1977 | hematuria | IgA, IgG, IgM, IgE, IgD | IgA concentration was significantly higher in patients with IgAVN (p<0.0125) |
| Song et al., 2022 | hematuria - >5rbc/hpf or positive dipstick (+ or ++ 3 consecutive days, +++ 2 consecutive days), proteinuria >300mg/24h or positive dipstick (+ or ++ 3 consecutive days, +++ 2 consecutive days), nephritic syndrome or kidney insufficiency | WBC, CRP, Fib, NLR, D-dimers, IgA, C3, C4 | NLR was significantly higher in moderate and severe IgAVN compared to patients without systemic involvement (p<0.0001), C3 was significantly decreased in severe IgAVN group compared to moderate IgAVN or no systemic involvement (p<0.0001) |
| Su et al., 2016 | hematuria and or proteinuria | IL-2, IL-4, IL-6, IL-10, IL-17A, TNF, IFNg, midkine (MK) | MK elevation was higher in IgAVN group compared to IgAV (p<0.05) |
| Sun et al., 2017 | hematuria - macroscopic or microscopic >5rbc/hpf, proteinuria - 3 positive dipsticks within a week or microalbuminuria | FOBT, WBC, Ne, PLT, MPV, CysC, CRP, D-dimers | positive FOBT (p=0.033), elevated CRP (p=0.022) and elevated D-dimers (p=0.00) are more frequent in IgAVN patients |
| Sunar et al., 2023 | hematuria - red cells casts, >5rbc/hpf, 2+ or higher on dipstick, proteinuria - >4mg/m2/h | CRP | CRP elevation was associated with the risk of steroid-dependent disease (p=0.017) |
| Tanaka H et al., 2023 | hematuria - gross hematuria or hematuria with more than 3 RBC/high-power field 3 times within 1 week, proteinuria - 3 positive dipsticks in a week/ more than 150mg/24h, UACR over 0,2 mg/mg, elevated uriNAry microalbumin more than 3 times within 1 week | machine learning prediction model including EB-DNA, WBC, Alb, Cr, TG, TC - 4 models: logistic regression, support vector machine, decission tree, random forest | Random forest model has the highest precision rate, accuracy rate, recall rate and F1 score (0.83; 0.87; 0.86; 0.85); AUC - 0.912 |
| Tang et al., 2021 | proteinuria and/or hematuria | C3, C4, IgA, IgG, IgM, IgE, Gd-IgA1 | IgA (p=0.011), and IgM (p=0.003) were higher in patients with IgAVN, IgG (p=0.001) was lower in patients with IgAVN |
| Ture et al., 2021 | proteinuria >300mg/24h, UACR > 30mmol/mg, dipstick >2+ | urinary KIM-1, urinary NGAL | KIM-1 (p=0.001) and KIM-1/creatinine ratio (p=0.006), as well as NGAL (p=0.005) and NGAL/creatinine ratio (p=0.008) are significantly higher in patients with IgAVN |
| Wang B et al., 2020 | NA | IgG, IgA, IgM, C3, C4, IgA1, Gd-IgA1, folicular T helper cells (Tfh), IL-21, IL-6 | Tfh, IL-21, IL-6, IgA1, Gd-IgA1 are significantly higher in IgAVN patients (p<0.05) |
| Wang J et al., 2017 | hematuria - >5rbc/hpf or red blood cells casts, proteinuria - 0.3g/24h | urinary MCP-1 | urinary MCP-1 levels were significantly higher in IgAVN group (p<0.001) |
| Wang J et al., 2017 | hematuria - more than 5rbc/hp field, proteinuria - dipstick (+) ormore than 4mg/m2/h on 24h samples | serum levels of insulin like growth factor 1 (IGF-1) and IGFBP-3; C3, C4, CRP, Hb, PLT, WBC | serum levels of both markers were higher in patients with proteinuria compared to those without (p-0.001, p-0.005); did not differ between patients with or without hematuria, p>0.005; c3, C4, CRP, Hb, PLT, WBC did not differ between the patients with and without hematuria and/or proteinuria |
| Wang J et al., 2022 | hematuria - gross hematuria or more than 5 rbc/hp field, proteinuria - at least 3 positive dipsticks within 1 week, more than 150mg proteinuria/24h, increased microalbuminuria at least 3 times within 1 week | urinary MIF level (macrophage migration inhibitory factor) | urinary MIF levels are significantly increased in IgAVN patients compared to IgAV and controls (p<0.001) |
| Wang J et al., 2023 | hematuria - macroscopic or microscopic (3 times within 1 week) > 3rbc/hpf, proteinuria (3 times within 1 week) >150mg/24h or UPCR>0.2 mg/mg, microalbuminuria | vitamin D levels | vitamin D levels are significantly decreased in IgAVN patients (p=0.00) |
| Wright et al., 2022 | UACR > 30mg/mmol | Urinary C3/creatinine ratio, C4/creatinine ratio, C5/creatinine ratio, C5a/creatinine ratio | Urinary C3/creatinine ratio, C4/creatinine ratio, C5/creatinine ratio, C5a/creatinine ratio can distinguish between patients with and without IgAVN (AUC; C3 – 0.085,p= 0.0009; C4 – 0.805,p= 0.0033; C5 – 0.838,p= 0.0012; and C5a– 0.817,p= 0.0024) |
| Wu et al., 2019 | hematuria and or proteinuriia | tryglicerides (TG), Chol, HDL-C, LDL-C, apoA, apoB, apoM, uric acid, hs-CRP, IgG, IgM, IgA, IgE, C3, C4 | UA is significantly higher in patients with IgAVN (p<0.05), IgG and C4 levels are significantly lower in patients with IgAVN (p<0.05), apoM (OR=0.37, 95%95%CI 0.156-0.877) was identified as a protective factor for IgAVN |
| Xi et al., 2021 | hematuria - >5rbc/hpf or >2+ dipstick, proteinuria >0.3g/24h | WBC, Ly, Ne, Eo, Mo, RBC, Hb,PLT, MCV, MCH, MCHC, RDW-SD, RDW-CV, PCT, MPV, PDW, CRP, NLR, PLR, IgG, IgA, IgM, IgE, C3, C4, APTT, PT, Fib, D-dimers, Total proteins (TP), Alb, AST, ALT, CD3+, CD4+, CD8+ | WBC (p=0.03), NLR (p=0.039), MCHC (p=0.014), CRP (p=0.01), IgG (p=0.003), TP (p=0.013), Alb (p=0.029) are significantly lower in patients with IgAVN, Eo (p=0.046), MPV (p=0.008), PDW (p=0.002), Fib (p=0.002), CD3+ (p=0.002), CD4+ (p=0.02) are significantly higher in patients with IgAVN |
| Xu et al., 2017 | hematuria - >5rbc/hpf, proteinuria >150mg/24h | WBC, Ne, Hb, MCV, PLT, MPV, RDW, CRP, ESR, Chol | IgAVN patients exhibited higher levels of Hb (p<0.001), MCV (p=0.007), MPV (p<0.001), RDW (p<0.001), Chol (p<0.001) and lower levels of PLT (p<0.001) and CRP (p<0.001) |
| Yakut et al., 2020 | hematuria - >5rbc/hpf, proteinuria - UPCR >0.2 in children >2y | Hb, WBC, PLT, NLR, MPV, ESR, CRP | MPV was significantly higher in patients with IgAVN (p=0.027) |
| Yeganeh et al., 2024 | NA | serum and urinary Ang-1, Ang-2, Tie2 (angiopoietins) | Ang1/Ang2 reduced in IgAV |
| Yildiz B et al., 2008 | hematuria and or proteinuria | f XIII activity | no significant difference between patients with and without IgAVN, but in patients with IgAVN, those with lower fXIII activity had more severe proteinuria (p< 0.05) |
| Yoshioka et al., 1980 | hematuria and or proteinuria | metabolomics analysis of urine samples (LC-MS/MS analysis) - differentially expressed metabolites | establishes a combined predictive model consisting of propionylcarnitine and indoxyl sulfate for IgAVN in HSP patients. |
| Yu et al., 2024 | hematuria and or proteinuria | ESR, C3, C4, ASOT, Igs A,M,G,E, CD3+ cells, CD4+ cells, CD3-CD16+CD56+ cells, CD4+/CD8+ cells | increase of cd3+cd8+ cells and normal cd3+ cells were positively correlated with IgAVN (p-0.032; p-0.014) |
| Yu S et al., 2024 | hematuria >5rbc/hpf, proteinuria > 300mg/24h | plasma IgA, IgA-Ics, sCD89-IgA | elevated plasma sCD89-IgA levels are a high risk factor for IgAVN (p=0.027, OR=1.015, 95%CI=1.002-1.028) |
| Yuan et al., 2018 | NA | TNFa, IL-8, IL-10 | TNFa level was significantly higher in the IgAVN group (p < 0.05) |
| Yuksel et al., 2015 | hematuria and or proteinuria | vitamin D levels | vitamin D levels are significantly decreased in IgAVN patients (p<0.05) |
| Zhang J et al., 2015 | hematuria and or proteinuria | CRP, procalcitonin, RBC, Hb, WBC, Ne, Ly, Mo, Eo, PLT, MPV, NLR, MLR, PLR, PT, APTT, D-dimers, FDP | Hb is significantly lower in patients with IgAVN (p=0.003), while D-dimers are significantly higher in patients with IgAVN (p=0.048) |
| Zhang N et al., 2017 | biopsy proven IgAVN or proteinuria over 150mg/24h | urine proteomics analysis - mass spectometry diaPASEF | increased level of AZGP-1in patients with IgAVN (p< 0.05) |
| Zhang Y et al., 2017 | NA | WBC, PCT, ESR, IgA, IgG, IgM, C3, ASOT | WBC increased is correlated with IgAVN (p=0.018) |
| Zhang Y et al., 2022 | NA | urinary KIM-1, Albumin, urinary NAG | the urinary KIM-1 and NAG are significantly increased in patients with IgAVN (p<0.05), while the serum albumin level is significantly decreased in IgAVN patients (p=0.019) |
| Zhao et al., 2019 | NA | RDW-CV, Hb, WBC, PLT, ESR, IgG, IgM, IgA | RDW-CV was higher in IgAVN patients (cut-off 14% - sensitivity 71,1%, specificity 97,9%, p<0.001) |
| Zhu L et al., 2022 | hematuria - macroscopic or microscopic >5rbc/hpf or >+ dipstick, proteinuria > 4mg/m2/h or >+ dipstick | WBC, Hb, CRP, ESR | no statistically significant difference between patients with and without IgAVN |
| Zhu X et al., 2017 | NA | Eo, Ne, Ly, PLT, RDW-CV, ENR, NLR, PLR | ENR>2.6 (OR=4.305 95%CI=1.11-16.63), PLR>104 (OR=5.935 95%CI=1.26-29.91), Ne > 9.1 (OR=7.761 95%CI = 2.02-29.88) were identified as significant risk factors for IgAVN |
| Zhu X et al., 2023 | hematuria and/or proteinuria | LTB4, vitamin D | LTB4 level was significantly higher and vitamin D levels were significantly lower in IgAVN patients (p<0.05) |
| Zhu Z et al., 2023 | hematuria and or proteinuria | PTX3, Hb, WBC, PLT, IgA, IgM, IgG, IgE, CRP, ESR, ASOT, RF, Fib, C3, C4 | the mean serum level of PTX3 was significantly higher in patients with IgAVN (p=0.004) |

**Results of Hematological Biomarkers**

Figure S2. MDs of WBC in patients with IgAV with and without kidney involvement by country

Figure S3. ORs of having an elevated WBC in patients with IgAV with and without kidney involvement by country

Figure S4. Diagnostic forest plot for WBC

Figure S5. MDs of Ne count in patients with IgAV with and without kidney involvement by country

Figure S6. MDs of Ne percentages in patients with IgAV with and without kidney involvement

Figure S7. ORs of having an elevated Ne count in patients with IgAV with and without kidney involvement

Figure S8. MDs of Ly counts in patients with IgAV with and without kidney involvement by country

Figure S9. MDs of Mo counts in patients with IgAV with and without kidney involvement

Figure S10. MDs of Eo counts in patients with IgAV with and without kidney involvement

Figure S11. MDs of RBC in patients with IgAV with and without kidney involvement

Figure S12. MDs of Hb in patients with IgAV with and without kidney involvement by country

Figure S13. ORs of having a decreased Hb in patients with IgAV with and without kidney involvement

Figure S14. Diagnostic forest plot for Hb

Figure S15. MDs of MCV in patients with IgAV with and without kidney involvement

Figure S16. ORs of having a decreased MCV in patients with IgAV with and without kidney involvement

Figure S17. MDs of RDW-CV in patients with IgAV with and without kidney involvement

Figure S18. MDs of RDW-SD in patients with IgAV with and without kidney involvement

Figure S19. MDs of PLT counts in patients with IgAV with and without kidney involvement by country

Figure S20. ORs of having an elevated PLT count in patients with IgAV with and without kidney involvement by country

Figure S21. Diagnostic forest plot for PLT

Figure S22. MDs of plateletcrit in patients with IgAV with and without kidney involvement

Figure S23. MDs of PDW in patients with IgAV with and without kidney involvement

Figure S24. MDs of MPV in patients with IgAV with and without kidney involvement by country

Figure S25. ORs of having an elevated MPV in patients with IgAV with and without kidney involvement

Figure S26. MDs of MPR levels in patients with IgAV with and without kidney involvement

Figure S27. ORs of having an elevated NLR in patients with IgAV with and without kidney involvement

Figure S28. ORs of having an elevated PLR in patients with IgAV with and without kidney involvement

**Results of Coagulation Biomarkers**

Figure S29. MDs of APTT levels in patients with IgAV with and without kidney involvement

Figure S30. SMDs of D-dimers levels in patients with IgAV with and without kidney involvement by country

Figure S31. ORs of having an elevated D-dimers level in patients with IgAV with and without kidney involvement

Figure S32. SMDs of Fibrinogen levels in patients with IgAV with and without kidney involvement

**Results of Inflammatory Biomarkers**

Figure S33. MDs of CRP levels in patients with IgAV with and without kidney involvement by country

Figure S34. ORs of having an elevated CRP in patients with IgAV with and without kidney involvement by country

Figure S35. Diagnostic forest plot for CRP

Figure S36. SMDs of ESR levels in patients with IgAV with and without kidney involvement by country

Figure S37. ORs of having an elevated ESR in patients with IgAV with and without kidney involvement by country

Figure S38. Diagnostic forest plot for ESR

Figure S39. SMDs of IL-6 levels in patients with IgAV with and without kidney involvement

Figure S40. SMDs of IL-8 levels in patients with IgAV with and without kidney involvement

Figure S41. SMDs of IL-10 levels in patients with IgAV with and without kidney involvement

Figure S42. SMDs of TNFα levels in patients with IgAV with and without kidney involvement

**Results of Immunological Biomarkers**

Figure S43. Median differences of ASOT levels in patients with IgAV with and without kidney involvement

Figure S44. ORs of having a positive ASOT in patients with IgAV with and without kidney involvement

Figure S45. Diagnostic forest plot for ASOT

Figure S46. SMDs of C3 levels in patients with IgAV with and without kidney involvement by country

Figure S47. ORs of having a decreased C3 level in patients with IgAV with and without kidney involvement

Figure S48. ORs of having an elevated C3 level in patients with IgAV with and without kidney involvement

Figure S49. Diagnostic forest plot for C3

Figure S50. SMDs of C4 in patients with IgAV with and without kidney involvement by country

Figure S51. ORs of having a decreased C4 level in patients with IgAV with and without kidney involvement

Figure S52. ORs of having an elevated C4 level in patients with IgAV with and without kidney involvement

Figure S53. Diagnostic forest plot for C4

Figure S54. SMDs of IgA levels in patients with IgAV with and without kidney involvement by country

Figure S55. ORs of having an elevated IgA level in patients with IgAV with and without kidney involvement by country

Figure S56. Diagnostic forest plot for IgA

Figure S57. SMDs of IgE levels in patients with IgAV with and without kidney involvement

Figure S58. SMDs of IgG levels in patients with IgAV with and without kidney involvement by country

Figure S59. ORs of having an elevated IgG in patients with IgAV with and without kidney involvement

Figure S60. Diagnostic forest plot for IgG

Figure S61. SMDs of IgM levels in patients with IgAV with and without kidney involvement by country

Figure S62. ORs of having an elevated IgM level in patients with IgAV with and without kidney involvement

**Results of Kidney Injury-related Biomarkers**

Figure S63. MDs of Albumin levels in patients with IgAV with and without kidney involvement by country

Figure S64. ORs of having a decreased Albumin level in patients with IgAV with and without kidney involvement

Figure S65. Diagnostic forest plot for Albumin

Figure S66. SMDs of cholesterol levels in patients with IgAV with and without kidney involvement

Figure S67. SMDs of CysC levels in patients with IgAV with and without kidney involvement

Figure S68. SMDs of urinary β_2_ MG levels in patients with IgAV with and without kidney involvement

**Results of Miscellaneous Biomarkers**

Figure S69. SMDs of endothelin-1 levels in patients with IgAV with and without kidney involvement

Figure S70. SMDs of vitamin D levels in patients with IgAV with and without kidney involvement

Figure S71. Diagnostic forest plot for FOBT

Figure S72. Funnel plot assessing the risk of publication bias for the PLT (MD) outcome

Figure S73. Funnel plot assessing the risk of publication bias for the WBC (MD) outcome

**Risk of bias assessment results**

Figure S74. Risk of bias assessment using Quality in Prognostic Studies (QUIPS) tool: Summary Plot

Table S4. Risk of bias assessment using Quality in Prognostic Studies (QUIPS) tool: part 1

Table S5. Risk of bias assessment using Quality in Prognostic Studies (QUIPS) tool: part 2

Table S6. Risk of bias assessment using Quality in Prognostic Studies (QUIPS) tool: part 3

Table S7. Risk of bias assessment using Quality in Prognostic Studies (QUIPS) tool: part 4

**References**

1.	Luo D, Wan X, Liu J, Tong T. Optimally estimating the sample mean from the sample size, median, mid-range, and/or mid-quartile range. *Statistical Methods in Medical Research* 2018; **27**(6): 1785-805.

2.	Shi J, Luo D, Weng H, et al. Optimally estimating the sample standard deviation from the five-number summary. *Res Synth Methods* 2020; **11**(5): 641-54.

3.	Knapp G, Hartung J. Improved tests for a random effects meta-regression with a single covariate. *Stat Med* 2003; **22**(17): 2693-710.

4.	IntHout J, Ioannidis JP, Borm GF. The Hartung-Knapp-Sidik-Jonkman method for random effects meta-analysis is straightforward and considerably outperforms the standard DerSimonian-Laird method. *BMC Med Res Methodol* 2014; **14**: 25.

5.	Hedges LV. Distribution Theory for Glass's Estimator of Effect size and Related Estimators. *Journal of Educational Statistics* 1981; **6**(2): 107-28.

6.	Mathias Harrer PC, Toshi Furukawa, David Ebert. Doing Meta-Analysis with R: A Hands-On Guide. 1st ed. New York: Taylor & Francis; 2021.

7.	Veroniki AA, Jackson D, Viechtbauer W, et al. Methods to estimate the between-study variance and its uncertainty in meta-analysis. *Res Synth Methods* 2016; **7**(1): 55-79.

8.	Chu H, Cole SR. Bivariate meta-analysis of sensitivity and specificity with sparse data: a generalized linear mixed model approach. *J Clin Epidemiol* 2006; **59**(12): 1331-2; author reply 2-3.

9.	Freeman SC, Kerby CR, Patel A, Cooper NJ, Quinn T, Sutton AJ. Development of an interactive web-based tool to conduct and interrogate meta-analysis of diagnostic test accuracy studies: MetaDTA. *BMC Med Res Methodol* 2019; **19**(1): 81.

10.	Lee J, Kim KW, Choi SH, Huh J, Park SH. Systematic Review and Meta-Analysis of Studies Evaluating Diagnostic Test Accuracy: A Practical Review for Clinical Researchers-Part II. Statistical Methods of Meta-Analysis. *Korean J Radiol* 2015; **16**(6): 1188-96.
